# Comparative authentication of *Hypericum perforatum* herbal products using DNA metabarcoding, TLC and HPLC-MS

**DOI:** 10.1038/s41598-017-01389-w

**Published:** 2017-05-02

**Authors:** Ancuta Cristina Raclariu, Ramona Paltinean, Laurian Vlase, Aurélie Labarre, Vincent Manzanilla, Mihael Cristin Ichim, Gianina Crisan, Anne Krag Brysting, Hugo de Boer

**Affiliations:** 1 Natural History Museum, University of Oslo, P.O. Box 1172 Blindern, 0318 Oslo, Norway; 2 NIRDBS/”Stejarul” Research Centre for Biological Sciences, Alexandru cel Bun Street, 6, 610004 Piatra, Neamt Romania; 3 Department of Pharmaceutical Botany, University of Medicine and Pharmacy “Iuliu Haţieganu”, Faculty of Pharmacy, Gheorghe Marinescu Street, 23, 400337 Cluj-Napoca, Romania; 4 Department of Pharmaceutical Technology and Biopharmaceutics, “Iuliu Hatieganu” University of Medicine and Pharmacy, Ion Creanga Street, 8-10, 400010 Cluj-Napoca, Romania; 5 Department of Biosciences, Centre for Ecological and Evolutionary Synthesis (CEES), University of Oslo, P.O. Box 1066 Blindern, 0316 Oslo, Norway

## Abstract

Many herbal products have a long history of use, but there are increasing concerns over product efficacy, safety and quality in the wake of recent cases exposing discrepancies between labeling and constituents. When it comes to St. John’s wort (*Hypericum perforatum* L.) herbal products, there is limited oversight, frequent off-label use and insufficient monitoring of adverse drug reactions. In this study, we use amplicon metabarcoding (AMB) to authenticate 78 *H*. *perforatum* herbal products and evaluate its ability to detect substitution compared to standard methods using thin-layer chromatography (TLC) and high performance liquid chromatography coupled with mass spectrometry (HPLC-MS). *Hypericum perforatum* was detected in 68% of the products using AMB. Furthermore, AMB detected incongruence between constituent species and those listed on the label in all products. Neither TLC nor HPLC-MS could be used to unambiguously identify *H*. *perforatum*. They are accurate methods for authenticating presence of the target compounds, but have limited efficiency in detecting infrageneric substitution and do not yield any information on other plant ingredients in the products. Random post-marketing AMB of herbal products by regulatory agencies could raise awareness among consumers of substitution and would provide an incentive to manufacturers to increase quality control from raw ingredients to commercialized products.

## Introduction

St. John’s wort (*Hypericum perforatum* L.) herbal products are popular in complementary and alternative medicine, and are widely used to treat mild to moderate depression but have a much broader traditional use^1^. These products play an important role in primary healthcare, and their popularity is determined by consumer health concerns, cultural habits and by the belief that they are natural and thus safe^2^. *Hypericum perforatum* is among the top-selling herbs and is sold as over-the-counter (OTC) products in pharmacies, supermarkets, health shops and through e-commerce^3^. Products are typically labeled as natural foods or dietary supplements, and claims regarding their possible health benefits appear on labels and in associated advertising. In 2011, the global market for herbal products was estimated to be US$83 billion^4^ with Europe being the largest market.

Lack of standardized methods for quality assessment and the highly competitive market of herbal products has increased the incentive to use substitutes and unlabeled fillers^5–7^. However, adulteration is not necessarily intentional, and herbal products may be altered due to accidental adulteration, misidentification^8^ and confusion resulting from vernacular names^9, 10^. In any case, the use of unreported ingredients is a serious safety concern as adverse drug reactions cannot be associated to the product label and ingredients^11, 12^. Signal detection in herbal adverse drug reactions is greatly impeded if ingredients in the products are not reported on the labels^10^.

The European Medicines Agency (EMA) is the European Union agency that is responsible for the evaluation of medicinal products. However, differences in herbal medicine classification exist between EU/EEA member states and this complicates quality monitoring of these products^11^. EMA does not test the composition of herbal products or verify whether ingredients included on the label are included in the product but delegates this responsibility to the manufacturers of these products. EMA requires quality assurance of herbal substances, preparations and products and specifies the use of macroscopic and microscopic characterization, phytochemical analysis of therapeutic target compounds and markers and assays for toxic constituents such as heavy metals and toxins^13^. EMA suggests that identification tests specific for substitute and adulterant detection either use a combination of separate chromatographic approaches (e.g., HPLC with TLC-densitometry) or combine different approaches into a single procedure (e.g., HPLC-UV, HPLC-MS or GC-MC)^13^. Herbal products are usually highly processed and have numerous ingredients, and applying these methods might not enable the accurate identification of all plant ingredients, especially if target species are admixed with other species within the same genera. To complement traditional identification methods, the EMA, as well as the United States Food and Drug Administration (FDA), support the use of innovative analytical technologies such as DNA barcoding.

DNA barcoding is a validated molecular identification method that can provide species-level resolution that is commonly used in authentication of taxonomic provenance of herbal products^5–7, 14^. Sanger sequencing based DNA barcoding studies have revealed widespread levels of substitution: 6% in saw palmetto herbal dietary supplements^15^, 16% in ginkgo products^16^, 25% in black cohosh^17^, 33% in herbal teas^18^, and 50% in ginseng^19^. A blind test of 44 herbal products sold in North America using DNA barcoding^7^ found that 59% contained species not listed on the labels, and only two out of twelve screened companies had products free of substitution, contamination or unreported fillers^7^. High-throughput sequencing based amplicon metabarcoding (AMB)^20^ studies can provide insights into species composition of complex mixtures of DNA such as processed herbal products. For example, in a study by Coghlan *et al*.^5^, the species composition of 15 highly processed traditional Chinese medicines (TCM) were evaluated using high-throughput sequencing and found that these contained species and genera included on CITES appendices I and II. Other AMB studies have shown similar concerns of varying quality and product label-content fidelity. For instance, Ivanova *et al*.^21^ found that 15 tested herbal supplements contained non-listed, non-filler plant DNA and Cheng *et al*.^22^ showed that the quality of 27 tested herbal preparations was highly affected by the presence of contaminants.

In this study, we investigated complex herbal products containing St. John’s wort *(Hypericum perforatum*), marketed in the EU/EEA both as herbal food supplement and herbal drug. St. John’s wort has traditional indications in nervous system, psychiatric, gastrointestinal, hepatobiliary, renal and urinary, respiratory, thoracic, endocrine, musculoskeletal, metabolism and nutritional disorders, as well as in infections and infestations^23^. The mode of action of the major responsible bioactive compounds of *H*. *perforatum* is still not completely known, but it seems that they act in a synergetic manner to achieve the clinical effectiveness^24^. However, several studies showed that the antidepressant activity is associated mainly to the phloroglucinol derivative hyperforin^25^ and the naphthodianthrones hypericin and pseudohypericin^26^. The quantity and quality of active constituents in *Hypericum* herbal medicines are highly affected by the manufacturing process^1^. Treatments involving St. John’s wort are generally safe, but several studies show that use in combination with other drugs can cause potentially life-threatening adverse drug reactions due to pharmacokinetic interactions^3^. These adverse interactions are still not fully understood despite extensive studies in its mechanisms. *Hypericum* extracts can cause serious side effects when administrated simultaneously with some antidepressants^27^. Other studies reported adverse reactions due to high doses of hypericin in *Hypericum* products leading to a phototoxic effect that may induce photodermatitis and can also decrease or nullify the effect of other drugs when administrated simultaneously^28^. Hall *et al*.^29^ reported exacerbated vaginal breakthrough bleeding, and Murphy *et al*.^30^ observed evidence of follicle growth and probable ovulation when simultaneously administrating *H*. *perforatum* with low-dose oral contraceptives.

The aim of this study was to evaluate the ability of using AMB to detect substitution in single and multi-ingredient *Hypericum* herbal products compared with standard identification approaches suggested by the European Pharmacopoeia and the European Medicine Agency, such as HPLC-MS and TLC.

## Results

### Thin Layer Chromatography

**Figure 1:**
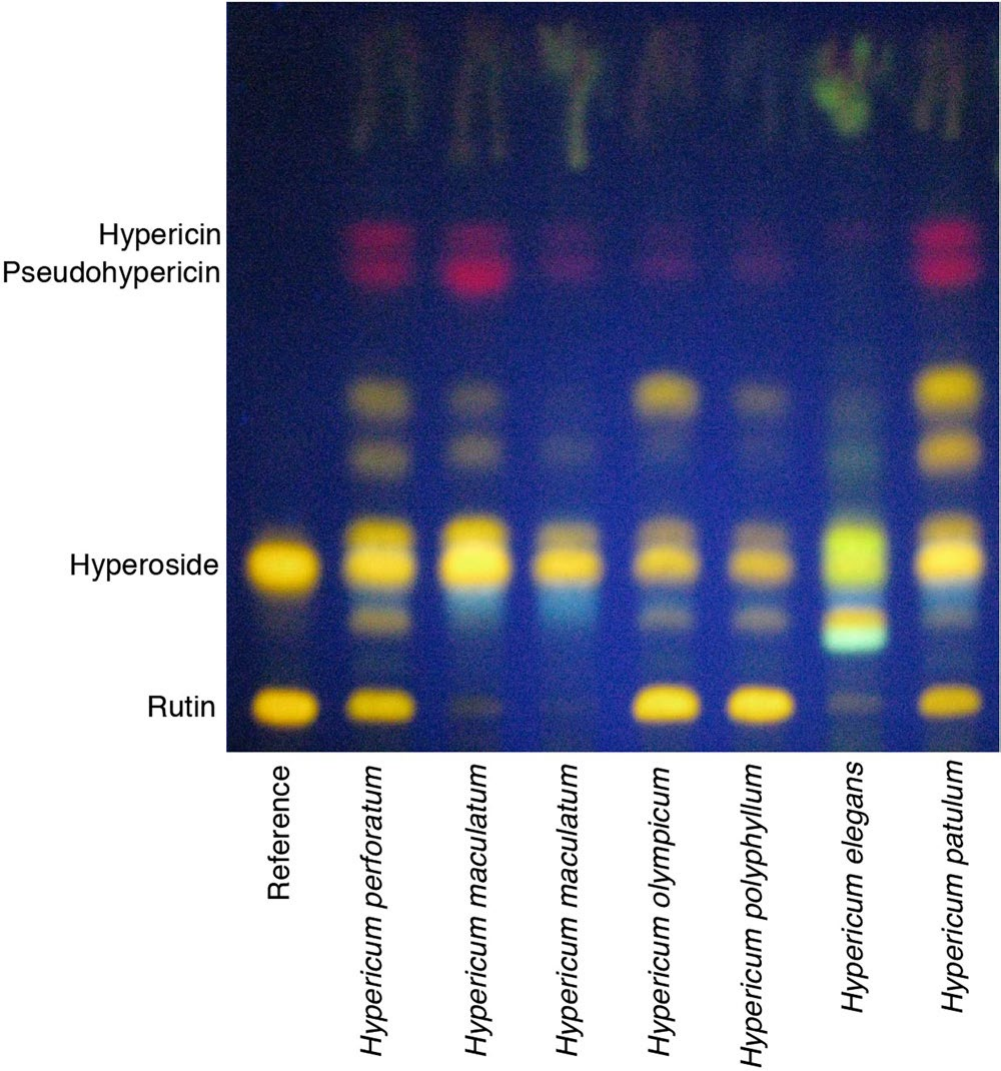
Thin layer chromatogram (TLC) of *Hypericum perforatum* and other *Hypericum* species. The yellow-orange fluorescent bands from the lower third of the chromatogram correspond to rutin and hyperoside, and are used for the identification of *H*. *perforatum*. This distinguishes between presence of rutin in *H*. *perforatum* and absence or only small quantities of rutin in *H*. *maculatum*. The bands corresponding to rutin and hyperoside are found also in *H*. *olympicum*, *H*. *patulum*, and *H*. *polyphyllum*.

The European Pharmacopoeia sets a minimum concentration of 0.08% total hypericins in the dried drug^31^, and includes a TLC based identification assay intended to distinguish *H*. *perforatum* from other species, including other species within the same genus. The European Pharmacopoeia 8.0^31^ *Hyperici herba* monograph TLC test yields four zones, corresponding to rutin, hyperoside, pseudohypericin and hypericin, calibrated by two reference solution compounds, rutin and hyperoside (Fig. 1). This can be used to distinguish between presence of rutin in *H*. *perforatum* and small quantities of rutin in *H*. *maculatum* Crantz. Our test control samples included *H*. *elegans* Stephan ex Willd., *H*. *maculatum*, *H*. *olympicum* L., *H*. *patulum* Thunb., *H*. *perforatum*, and *H*. *polyphyllum* Boiss. & Balansa. This testing revealed some remarkable challenges associated with this authentication method (Fig. 1). The chromatograms of *H*. *olympicum*, *H*. *patulum*, and *H*. *polyphyllum* were indistinguishable from those of *H*. *perforatum*.

**Figure 2:**
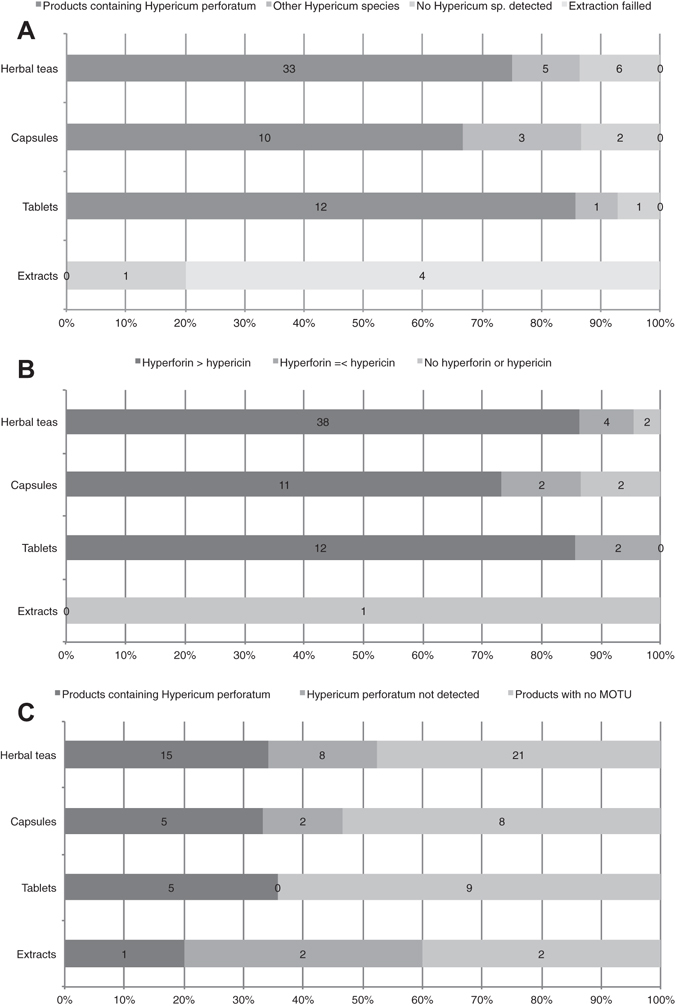
Presence of *Hypericum perforatum* within the products. (**A**) Detection using thin layer chromatogram (TLC). (**B**) Detection using high-performance liquid chromatography-mass spectrometry (HPLC-MS). (**C**) Detection using amplicon metabarcoding (AMB). Detection between methods is not fully comparable as the resolution of the approaches differs.

The product samples were all tested using the recommended test. Sample extraction for four samples (a tincture, two oils, and one juice) out of 78 failed. The test results could be grouped into three categories: (1) rutin, hyperoside, pseudohypericin and hypericin present. This could indicate presence of *H*. *perforatum* or other *Hypericum* species with indistinguishable chromatograms, e.g. *H*. *olympicum*, *H*. *patulum*, and *H*. *polyphyllum*; (2) hyperoside, hypericin, and pseudohypericin present, but rutin in low or undetectable concentrations. This made presence of *H*. *perforatum* or other *Hypericum* species with indistinguishable chromatograms unlikely, but could indicate presence of *H*. *maculatum* or other *Hypericum* species with indistinguishable chromatograms, for example *H*. *elegans*; (3) rutin detected or not, and hyperoside, pseudohypericin and hypericin not detected. This ruled out presence of *Hypericum* species. Fifty-five out of 74 (74%) samples contained *H*. *perforatum* or an indistinguishable adulterant, or a mixture of *Hypericum* species including *H*. *perforatum* or an indistinguishable adulterant. Nine out of 74 (12%) samples contained *Hypericum* species other than *H*. *perforatum* or indistinguishable adulterants, or a mixture of those other *Hypericum* species. Ten out of 74 (14%) samples did not contain *Hypericum* species in detectable amounts (Fig. 2A; Supplementary Fig. S1; Supplementary Table S2).

### High-Performance Liquid Chromatography-Mass Spectrometry

Detection of *H*. *perforatum* using HPLC-MS is based on the presence of the two main bioactive compounds in *Hyperici herba*, hyperforin and hypericin. The *Hyperici herba* monograph in the European Pharmacopoeia 8.0^31^ states that the dried drug should have a minimum content of total hypericins, expressed as hypericin, corresponding to 0.08% to verify the presence of *H*. *perforatum*. A literature review of 88 taxa of *Hypericum* with qualitative (74 taxa^32^) and quantitative (27 taxa^33^) measures of hyperforin and hypericin, together with the six test control species measured in this study (Supplementary Table S3), revealed that the hyperforin and hypericin content was not an accurate predictor of *H*. *perforatum* presence. *Hypericum perforatum* and its main adulterant *H*. *maculatum* had hypericin contents within similar ranges, but *H*. *maculatum* had very low content of hyperforin, <0.018%. However, several of the tested or reviewed species had both hyperforin and hypericin contents similar to those of *H*. *perforatum*, most notably *H*. *olympicum* and *H*. *polyphyllum*.

All 78 product samples were analyzed using HPLC-MS, except for four samples that could not be extracted (a tincture, two oils, and one juice). The tested products showed presence of rutin, hyperoside, pseudohypericin and hypericin in 55 out of 74 (74%) samples, which could indicate *H*. *perforatum* or other *Hypericum* species with indistinguishable chromatograms. In nine out of 74 (12%) samples hyperoside, hypericin, and pseudohypericin were present, but rutin was present in low or undetectable concentrations, which made presence of *H*. *perforatum* or other *Hypericum* species with indistinguishable chromatograms unlikely, but could indicate presence of *H*. *maculatum* or other *Hypericum* species with indistinguishable chromatograms, such as *H*. *elegans*, or a mixture of *Hypericum* species. In ten out 74 (14%) samples rutin was detected or not, and hyperoside, pseudohypericin and hypericin were not detected, which ruled out presence of *Hypericum* species. The test results could further be grouped into three categories: (1) hyperforin content higher than hypericin content. This could indicate presence of *H*. *perforatum* or other *Hypericum* species with high levels of hyperforin, e.g. *H*. *elegans*, *H*. *olympicum* or *H*. *polyphyllum*; (2) hyperforin content equal to or lower than hypericin content. This could indicate presence of *H*. *maculatum* or other species with low levels of hyperforin, such as *H*. *barbatum* Jacq., *H*. *hirsutum* L., *H*. *humifusum* L., *H*. *linarioides* Bosse, *H*. *richeri* Vill., *H*. *rumeliacum* Boiss. or *H*. *tetrapterum* Fr.; (3) no hyperforin or hypericin detected. This indicates that material from *Hypericum* species is absent or that these compounds are present in undetectable amounts. It should be noted that low levels of hyperforin could also indicate low content of *H*. *perforatum* in the tested product. Sixty-one out of 74 samples (82%) had hyperforin content higher than hypericin content; eight out of 74 (11%) had hyperforin content equal to or lower than hypericin content; and five out of 74 (7%) had no detectable hyperforin or hypericin (Fig. 2B; Supplementary Table S4).

### DNA metabarcoding

The DNA extracted from 78 samples was highly variable in quantity and quality. Fragment Analyzer measurements gave results for 47 (60%) samples, with DNA concentration ranging from 0.01 to 140 ng/μl. Thirty-one samples (40%) did not contain measurable DNA concentrations: 11 capsules, eight tablets, eight herbal teas and four extracts. PCR amplification reactions were performed for all 78 samples and amplicons were obtained from 76% of the samples for nrITS1 and 73% for nrITS2. The highest amplification rate was obtained for herbal teas (97% for nrITS1 and 88% for nrITS2), followed by capsules (62% for nrITS1 and 62% for nrITS2), tablets (43% for nrITS1 and 45% for nrITS2) and extracts (27% for nrITS1 and 40% for nrITS2). There was no significant correlation between sample total DNA concentration and nrITS amplicon concentration or between amplicon concentration and sequenced reads or bases (Supplementary Table S5; Supplementary Fig. S6).

The raw data consisted of 9,416,033 sequences, with an average of 60,359 sequences per sample for each marker. Sequencing success rates were 49%, respectively 44% (34/78 samples) for nrITS1 and 47% (37/78 samples) for nrITS2. A dataset consisting of 1,511,356 reads, fulfilling our trimming and filtering quality criteria, was obtained, including 737,010 nrITS1 and 774,346 nrITS2 reads (on average 19,395 nrITS1 and 20,377 nrITS2 reads per sample). Forty samples out of 78 samples (51%) yielded no molecular operational taxonomic units (MOTUs) for either nrITS1 or nrITS2 and are excluded from the results and discussion (2, 7, 9, 11–14, 16, 18–20, 22, 23, 25, 26, 28, 31, 33, 34, 36, 40, 41, 45, 47, 49, 52–54, 56–58, 60, 62, 63, 64, 66–70). These included 21 herbal teas, eight capsules, nine tablets and two extracts (Supplementary Table S7). The MOTU yielding samples included 23 herbal teas, seven capsules, five tablets and three extracts.

**Figure 3:**
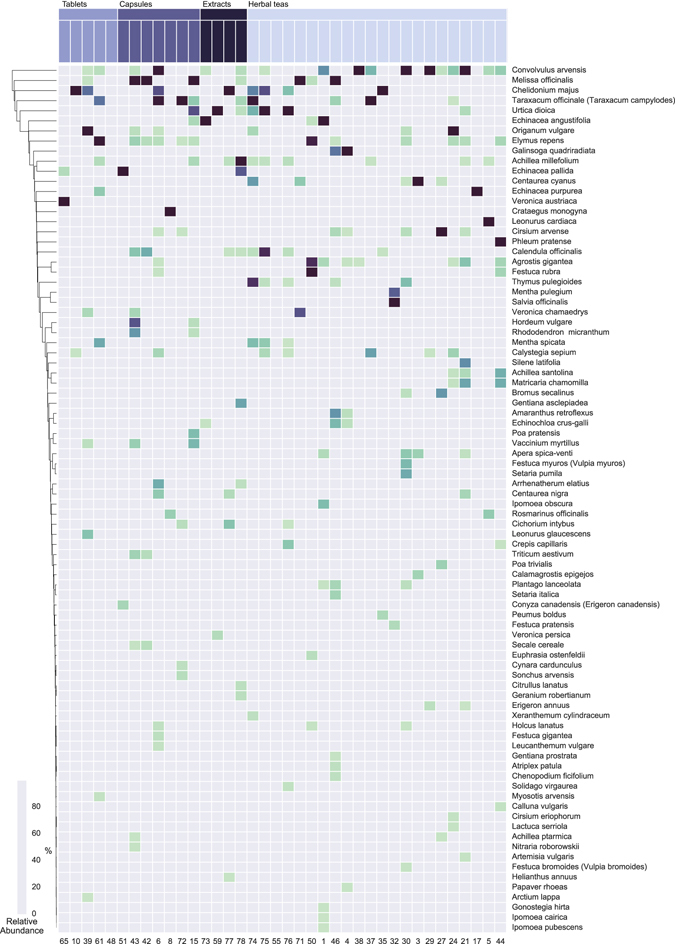
Species identified within the products using amplicon metabarcoding (AMB). Only MOTUs with >1% normalized read numbers per sample are shown. Species are colored according to relative abundance of normalized read numbers. Products are grouped by product form: herbal teas, capsules, tablets/pills/pastilles and extracts/tinctures/oils.

A total of 219 different species were identified using BLAST from the retained MOTUs (Supplementary Table S7; Fig. 3 for MOTUs with >1% normalized reads). For nrITS1 we detected a total of 143 different species and for nrITS2 137 species (Supplementary Fig. S8, Supplementary Fig. S9). Analysis of the BLAST identified MOTUs showed that a total of 34 genera (24%) and 76 species (36%) were detected exclusively with nrITS1. Analysis of nrITS2 showed that 82 (39%) species, but no unique genera, were detected exclusively with this marker. A total of 106 genera (76%) and 55 (26%) species were detected with both markers. Identified MOTUs and their read numbers were merged for both markers per sample for further analysis.

The number of species detected per sample ranged from one to 57, with an average of 18.7 species per sample. Out of the 17 successfully analyzed single ingredient samples, those containing only *H*. *perforatum* according to the label, none contained only *H*. *perforatum*, 13 contained more than one species, and four did not contain *H*. *perforatum*. Out of the 21 successfully analyzed multiple ingredient samples, those containing *H*. *perforatum* and other species according to the label, none contained only the ingredients listed on the label, two contained all species listed on the label plus additional species not listed on the label, 19 contained fewer species than listed on the label plus additional species, and eight did not contain *H*. *perforatum* (Fig. 2). The fidelity for *H*. *perforatum* in single ingredient products was 76% (13 out of 17), and for multi ingredient products, 62% (13 out of 21). The overall ingredient fidelity (detected species from product label/total number of species on label) for multi ingredient products was 41% and for all products 57%. The following four species were found in more than 50% of the samples: *Hypericum perforatum* (68%), *Convolvulus arvensis* L. (63%), *Achillea millefolium* L. (53%) and *Urtica dioica* L. (50%). Plant taxa present in more than 20% of the samples are listed in Table 1. Eleven out of the 78 products were commercialized and registered as herbal medicines, and 4 of these yielded AMB results. *Hypericum perforatum* was found in all four products, and in addition an average of 20.3 other species.

**Table 1:** Plant species detected by amplicon metabarcoding (AMB) in more than 20% of the products.

Species	Family	Occurrence
*Hypericum perforatum* L.	Hypericaceae	68%
*Convolvulus arvensis* L.	Convolvulaceae	63%
*Achillea millefolium* L.	Compositae	53%
*Urtica dioica* L.	Urticaceae	50%
*Elymus repens* (L.) Gould	Poaceae	47%
*Taraxacum campylodes* G.E.Haglund	Compositae	47%
*Melissa officinalis* L.	Lamiaceae	39%
*Calendula officinalis* L.	Compositae	37%
*Chelidonium majus* L.	Papaveraceae	37%
*Cirsium setidens* (Dunn) Nakai	Compositae	34%
*Mentha spicata* L.	Lamiaceae	29%
*Plantago lanceolata* L.	Plantaginaceae	29%
*Sambucus nigra* L.	Adoxaceae	29%
*Calystegia sepium* (L.) R. Br.	Convolvulaceae	26%
*Origanum vulgare* L.	Lamiaceae	26%
*Agrostis gigantea* Roth	Poaceae	24%
*Thymus pulegioides* L.	Lamiaceae	24%
*Viola tricolor* L.	Violaceae	24%
*Centaurea cyanus* L.	Compositae	21%
*Cichorium intybus* L.	Compositae	21%
*Cynara cardunculus* L.	Compositae	21%
*Echinacea angustifolia* DC.	Compositae	21%
*Hypericum humifusum* L.	Hypericaceae	21%
*Vaccinium myrtillus* L.	Ericaceae	21%

In addition to the target species, *H*. *perforatum*, that was detected in 68% (26) of the MOTU yielding samples, several other *Hypericum* species were also detected: *H*. *humifusum* in 21% (8), *H*. *tetrapterum* in 13% (5) and *H*. *hirsutum* in 3% (1). Other *Hypericum* species were never detected without *H*. *perforatum* suggesting that adulteration by admixture is more widespread than complete substitution. The five most common species detected with AMB, but which were not present on the label of the products were: *Agrostis gigantea* Roth (Poaceae) detected in 24%, *Centaurea cyanus* L. (Compositae) in 21%, *Vaccinium myrtillus* L. (Ericaceae) in 21%, *Lolium perenne* L. (Poaceae) in 18%, and *Apera spica-venti* (L.) P. Beauv. (Poaceae) in 16% of the samples. A total of 34 anemophilous (wind-pollinated) species were detected, including 31 grasses and the woody species *Fraxinus excelsior* L., *Humulus lupulus* L., and *Juglans regia* L. (Supplementary Table S7).

### Comparative results

In Fig. 2 the detection rates of the three authentication methods are summarized. The detection rate of AMB was much lower than that of the other approaches, but neither TLC nor HPLC-MS could be used to unambiguously identify *H*. *perforatum*, and it was thus difficult to make an overall comparison of the three methods. Considering only the 26 samples in which *H*. *perforatum* was detected using AMB, TLC could be used to detect rutin, hyperoside, hypericin and pseudohypericin in 21 samples (81%), and HPLC-MS could be used to detect higher hyperforin than hypericin content in 23 samples (88%). All three methods were in agreement for 19 samples (73%). For the 12 samples that yielded MOTUs using AMB but in which *H*. *perforatum* was not detected, TLC could be used to detect rutin, hyperoside, hypericin and pseudohypericin in six samples (50%), and HPLC-MS could be used to detect higher hyperforin than hypericin content in six samples (50%). For five samples (42%), the results from TLC and HPLC-MS were in agreement.

## Discussion

Misidentification of *H*. *perforatum* and/or adulteration of products containing *H*. *perforatum* with other *Hypericum* species, and the common and hyperforin-less *H*. *maculatum* in particular, requires the use of accurate analytical methods for the quality control of herbal products of *H*. *perforatum*. Identification of *Hypericum* species is feasible using taxonomic identification keys, but recognition of species in the field is challenging and many *Hypericum* species have superficially similar morphology. Positive identification requires the researchers to study flower, leaf and stem morphology including diagnostic characters that might be absent early or late in the flowering season. Identification is further complicated as *H*. *perforatum* may occasionally hybridize with other species in the genus, resulting in hybrids that have intermediate morphology and secondary metabolite spectra^34, 35^. Eastern Europe is a significant source of *Hypericum* material for European herbal products^36^, and here material is mostly wild-harvested and several different species co-occur, which could easily lead to intentional and/or accidental picking of the different species.

Using the TLC based identification assay included in the European Pharmacopoeia on a limited number of reference species included to check the accuracy of the test, show that several species have indistinguishable chromatograms from that of *H*. *perforatum* (Fig. 1). In addition to the ambiguous results with regard to different *Hypericum* species, the test would show the same chromatogram for an admixture of *H*. *perforatum* and *H*. *maculatum* as for an unadulterated *H*. *perforatum* sample. In addition, the TLC assay does not provide useful information about the concentrations of the main bioactive compounds, hyperforin and hypericin.

An alternative to testing for indicator compounds using TLC is to use HPLC-MS to detect and measure the content of the main bioactive secondary metabolites. This method does not claim to distinguish *H*. *perforatum* from other *Hypericum* species. Reviews state that the hyperforin content in *H*. *perforatum* is 2.0–4.5%, and the hypericin content 0.1–0.15% in fresh material^37, 38^. This is supported by several recent studies that have found hyperforin and hypericin contents of 1.4% and 0.5%, respectively, in the flowers and of 0.4–1.4% and 0.015–0.17%, respectively, in the vegetative parts^39, 40^. Both the hyperforin and hypericin contents vary greatly between studies, probably based on the nature and quality of the analyzed plant material^41^. To check the accuracy of HPLC-MS for distinguishing *H*. *perforatum*, we analyzed seven samples representing six *Hypericum* species, and compiled literature on hyperforin and hypericin values from 27 species^33, 39, 40, 42^. The results show that the hyperforin and hypericin contents vary considerably between and within species (Supplementary Table S3). *Hypericum perforatum* and its main adulterant *H*. *maculatum* have hypericin contents within similar ranges, but *H*. *maculatum* has very low content of hyperforin, 0.004–0.018%. This compilation highlights the low predictive value of hyperforin and hypericin content in determining substitution in *H*. *perforatum* preparations. Several species have similar hyperforin and hypericin contents to *H*. *perforatum*, most notably *H*. *olympicum* and *H*. *polyphyllum*.

The categorization of hyperforin and hypericin content per product into three groups made it possible to conclude that five products out of 74 (7%) have no detectable hyperforin or hypericin, eight out of 74 (11%) have low content or absence of *H*. *perforatum*, and 61 out of 74 samples (82%) have spectra typical of *H*. *perforatum* but also several other species. The 74 analyzed samples had hypericin contents ranging from 0–0.03%, and none passed the minimum threshold set by the European Pharmacopoeia (Supplementary Table S4). Wurglics *et al*.^43^ showed that commercial products bought in Germany have total hypericin contents ranging from 0.16–0.30%, and thus exceeding the European Pharmacopoeia minimum. The reason for the discrepancy between these and our results is not entirely clear, especially as both have been measured using similar methodology, but could be a result of lower product quality in our study material. In summary, HPLC-MS is not an accurate method for detection of substitution, adulteration or admixture, but suitable for control of bioactive compound content in products, and thus important in quality control for consumer safety. A superior, but more cost-intensive approach, than TLC and HPLC-MS is NMR metabolomics that enables chemical fingerprinting encompassing a huge range of target molecules^44^.

Several studies have shown the resolution and efficacy of DNA metabarcoding^20^ for identifying plant species diversity in a range of products^5, 6, 21, 22, 45–47^. Comparative identification of processed food and pharmaceutical products is challenging as compared to substrates that can be used for morphological identification, such as pollen clumps^46, 48^ and pollen in honey^45^. The advantage of pharmaceutical products and traditional and complementary alternative medicines is that these have their putative contents printed on the package^5, 6, 21, 22^. Galimberti *et al*.^48^, Richardson *et al*.^46^, and Hawkins *et al*.^45^ used *rbc*L and *trn*H*-psb*A, and nrITS2 and *rbc*L, respectively, to analyze DNA from pollen in pollen grains and honey to investigate honey bee foraging preferences. Cheng *et al*.^22^ used amplicon metabarcoding to analyze nine traditional Chinese medicines (TCMs) and detected on average 4.8 species using nrITS2 and 2.8 using *trn*L. Coghlan *et al*.^5, 6^ analyzed TCMs for presence of both animal and plant ingredients and found over 68 plant families and eight vertebrate genera in these products. Ivanova *et al*.^21^ used universal nrITS primers and found a host of plant species in eight herbal supplements, as well as many fungi due to specificity of these primers in amplifying fungal nrITS. In this study, plant specific nrITS primers were used to amplify nrITS1 and nrITS2, and an average of 18.7 species were detected per sample. In addition, the presence of *H*. *perforatum* was detected in 26 out of 38 sequenced samples (68%). These findings corroborate previous results that amplicon metabarcoding is an effective way to investigate species composition in products that contain a mixture of DNA from different species. Looking at the subset of registered herbal medicines that yielded AMB data, all four were found to contain *H*. *perforatum*, and this was supported by TLC results for all four, and by HPLC results in only two cases.

The relatively low success rate (49%) after applying strict read quality and filtering criteria makes this method challenging to use for routine screening at this time. Ivanova *et al*.^21^ reported a slightly higher success rate with eight out of 15 samples (53%) for nrITS2 AMB of herbal products from *Echinacea*, *Gingko*, *Hypericum*, *Trigonella*, and *Valeriana*. Cheng *et al*.^22^ reported a 100% success rate for 30 individual samples of TCM, all of which were unprocessed crude drugs. The varying degrees of success probably reflect the quality and type raw material, but also the many details in the analysis that can be varied to optimize the results, roughly in order of significance: extraction procedures and purification, primers, markers, identification approach, clustering, MOTU thresholds, sequencing platform, filtering, quality thresholds and chimera removal, library preparation, and amplification protocols.

Quantifying contamination is important when focusing on the tolerated levels of foreign matter in herbal pharmaceuticals. Quantifying relative species abundances based on sequence read numbers from samples with unknown ingredients is hampered by several factors. Firstly, AMB relies on the availability of DNA, but plant DNA can be removed or highly degraded during the harvesting, drying, storage, transportation, and processing (e.g., mode of extraction, irradiation, ultraviolet light exposure, heat or pressure, filtration, extractive distillation or supercritical fluid extraction)^49^. Secondly, AMB is a PCR-based method, and variation in nrITS copy number, primer annealing, and amplification bias all influence the number of taxon-specific reads^50^. Thirdly, incomplete reference databases and sequences with incorrect species names, can render taxonomic identifications prone to uncertainties.

AMB detected the target species, *H*. *perforatum*, in 68% of the samples, but in addition other *Hypericum* species were detected, *H*. *humifusum* in 21% (8), *H*. *tetrapterum* in 13% (5) and *H*. *hirsutum* in 3% (1) of the samples. *Hypericum tetrapterum* belongs to the same taxonomic section as *H*. *perforatum* but is more closely related to *H*. *maculatum*, whereas *H*. *humifusum* and *H*. *hirsutum* belong to sections Oligostema and Hirtella s.l., respectively^51, 52^. These species can co-occur in natural habitats from which *H*. *perforatum* is wild crafted. These other *Hypericum* species were never detected without *H*. *perforatum* suggesting that adulteration by admixture is more widespread than complete substitution.

The overlooked species diversity through poor primer fit and amplification bias is difficult to quantify but some diversity is likely missed^53^. The detection of additional plant species, other that the ones from the label or those that can be expected as substitutes, contaminants or fillers, may be explained by (1) amplified PCR chimeras; (2) false-positive BLAST identifications due to incomplete or error-prone reference databases; or (3) presence of pollen from anemophilous (wind-pollinated) species. Thirty-four of the MOTUs belong to anemophilous species, including 31 grasses of which *Agrostis gigantea* Roth (Poaceae) was detected in 24% of samples, *Lolium perenne* L. (Poaceae) in 18%, and *Apera spica-venti* (L.) P.Beauv. (Poaceae) in 16%. DNA from pollen from these species can end up in the products through co-occurrence with the collected material in natural habitats as well as during other steps in the process chain.

## Conclusions

The metabarcoding results confirm that AMB can be used to test for the presence of *H*. *perforatum* and simultaneously to detect substitution, adulteration and/or admixture of other species. These results corroborate with previous results that show the usefulness of metabarcoding for use in complementing traditional methods of quality control for consumer safety^5, 6, 21, 22^. It should be emphasized however that the relatively low success rate of generating sequence reads per product makes this method challenging to use for routine screening, as only 49% of the tested products yielded sequences as compared to 95% of products that could be used for TLC and HPLC-MS analyses. Moreover, the high sensitivity of AMB in detecting everything from grass pollen on field-collected plants to plant dust left in production equipment requires a careful consideration of the concept of contamination. AMB results for herbal pharmaceutical authentication should be interpreted with a focus on presence and or absence of target species, i.e. the labeled ingredients, but alarms need not be raised over trace contaminations from species plausibly present in the cultivation, transport or production chain. However, the TLC and HPLC-MS results show that these methods are of limited applicability with regard to detecting species substitution, but may be used efficiently to detect target compounds. The clear advantage of HPLC-MS over TLC is the ability to quantify constituents and to screen for a vastly larger number of compounds. If product safety relies on threshold levels of specific bioactive compounds, absence of toxins, allergens and admixed pharmaceuticals, then chemical analysis methods are more relevant than DNA based composition analysis, but if product fidelity, species substitution or adulteration is suspected then the latter method outperforms in terms of resolution. Development of novel molecular markers and approaches for genomic barcoding are likely to increase the resolution of DNA barcoding in species-level identification. Several others have advocated the use of DNA barcoding and metabarcoding in herbal product authentication and herbal pharmacovigilance^5, 7, 9, 21, 22, 54–56^ and adoption of standards for quality control by regulatory agencies could raise product quality and increase consumer confidence.

## Methods

### Sample collection

Seventy-eight herbal products that included *Hypericum perforatum* according to the label were randomly purchased in European countries (and Turkey), including Romania (51), Germany (5), Poland (4), Turkey (4), Slovakia (3), Spain (2), UK (2), Austria (2), Czech Republic (1), France (1), Italy (1), Sweden (1) and the Netherlands (1). The samples were bought from pharmacies (44), herbal shops (25), super markets (2) or via e-commerce (7), and were sold as herbal teas (44), capsules (15), tablets (14) and extracts (5). According to the label information, the products included 38 single ingredient products, 33 products contained between two and ten ingredients and seven products contained more than ten ingredients. These medicinal products for scientific analyses were imported into Norway under Norwegian Medicines Agency license no. 16/04551–2. An overview of the samples including label information, but not the producer/importer name, lot number, expiration date or any other information that could lead to the identification of that specific product can be found in Supplementary Table S10.

For the phytochemical analysis, aerial parts of *H*. *elegans* (voucher ARNHM01He), *H*. *maculatum* (ARNHM01Hm), *H*. *maculatum* (92151 UMF CLUJ), *H*. *olympicum* (ARNHM01Ho), *H*. *patulum* (ARNHM01Hpa), *H*. *perforatum* (92141 UMF CLUJ) and *H*. *polyphyllum* (ARNHM01Hpo) were used as references for the identification and quantification of the main compounds. The reference species were selected to include the main adulterant, *H*. *maculatum* (two geographically isolated samples from Romania and Norway), species with known high levels of hyperforin and hypericin, *H*. *polyphyllum* and *H*. *olympicum*, as well as random species collected during fieldwork in Romania, *H*. *patulum* and *H*. *elegans*. All voucher specimens are deposited in the Herbarium of the Alexander Borza Botanical Garden (CL) of Babes-Bolyai University, Cluj-Napoca, Romania.

### Phytochemical analysis

#### Thin layer chromatography (TLC)

Samples were processed according to the *Hyperici herba* monograph in the European Pharmacopoeia 8.0^31^. Control solutions were prepared by mixing 500 mg of ground identified and vouchered material of selected *Hypericum* species with 10 ml of methanol (Analytical grade, Chimreactiv SRL, Romania) for 10 min at 60 °C. After cooling, the obtained solution was filtered. The reference solution was obtained by dissolving 5 mg of hyperoside (Analytical grade, Sigma-Aldrich) and 5 mg of rutin (Analytical grade, Sigma-Aldrich) in 5 ml of methanol. The test solutions of the *Hypericum* products were prepared in the same way as the control solution. Herbal products were processed depending on the pharmaceutical formulation and following the principles of the same extraction procedure, which were adapted to each pharmaceutical formulation. The analysis of the samples was performed in triplicate on SilicaGel plates (60 G F_254_, 20 × 20 cm, Merck), in twin bands of 10 mm, consisting of a 10 µl test sample and a 5 µl reference solution. As a mobile phase, a mixture of formic acid (Analytical grade, Nordic):distilled water:ethyl acetate (Analytical grade, Lachner) (6:9:90 V/V/V) was used. After migration of the principal components, the plates were dried at 100–105 °C for 10 min. Detection was performed in UV light, at 365 nm, after spraying with a mixture of 1% methanolic diphenylboryloxyetylamine (Sigma-Aldrich) and 5% methanolic polyethylene glycol 400 (Sigma-Aldrich) and 30 min incubation.

#### High-Performance Liquid Chromatography-Mass Spectrometry (HPLC-MS)

*Hypericum* extracts were prepared by adding 750 mg of powdered plant material of *Hypericum* species, *H*. *perforatum* and *H*. *maculatum*, to 15 ml methanol in a glass tube^31, 57^. The tubes were capped and agitated in the dark at 25 °C for 3 hours on a digital ceramic hotplate stirrer (Arec. X. Velp Scientifica). The extracts were filtered and diluted (1:100) in the mobile phase consisting of a mixture of 1 mM acetonitrile (Merck)/ammonium acetate (Merck) 45/50 (V/V) in double distilled, deionised water (Infusion Solution Laboratory of the University of Medicine and Pharmacy Cluj-Napoca - Romania), and 1 µl of the mixture was injected into the HPLC chromatographic system. Quantities of the herbal product test samples were individually adapted in order to identify and quantify the reference compounds and the extraction procedure was subsequently followed as described above for the references. The HPLC system used was an 1100 series Agilent Technologies model (Darmstadt, Germany) consisting of a G1312A binary pump, an in-line G1379A degasser, a G1329A autosampler, a G1316A column thermostat and an Agilent Ion Trap Detector 1100 SL. Chromatographic separation was performed on a Zorbax SB-C18 (50 mm × 2.1 mm i.d., 3.5 µm) column (Agilent Technologies) equipped with a Zorbax SB-C18 precolumn with the mobile phase above, at 45 °C with a flow rate of 0.6 ml/min. The detection of analytes was performed in triplicate in non-reactive MS^2^ mode for the quantification of hypericin (Hwi Analytik Gmbh) or in reactive MS^2^ mode for hyperforin (Sigma), negative ion ionisation, using an ion trap mass spectrometer equipped with an electrospray ionisation ion source (ESI): capillary +2500 V, nebulizer 40 psi (nitrogen), dry gas nitrogen at 8 l/min, dry gas temperature 350 °C.

Standard calibration curves were obtained by plotting the peak areas of standard concentrations of hypericin (10, 20, 50, 100, 200 and 500 ng/ml) and hyperforin (2, 4, 10, 20, 40 and 100 ng/ml) against their nominal concentrations. Two linear regression equations (R^2^ > 0.998) were obtained. Positive identification of the target compounds was performed by mass-spectrometry, and quantification of hypericin and hyperforin was based on peak area (RT, retention time of 1.1 and 2.3 min, respectively) in comparison with the standard curves.

### Genetic analysis

#### DNA extraction and quantification

Total DNA was extracted using a modified CTAB extraction method from small amounts of each herbal product (about 300 mg)^58^. The substrate was homogenized using 2–3 zirconium grinding beads in a Mini-Beadbeater-1 (Biospec Products Inc., USA). The final elution volume was 100 μl, and extracted DNA was quantified using a Fragment Analyzer™ (Advanced Analytical Technologies, Inc., USA) and a DNF-488-33 HS Genomic DNA Reagent Kit (50 bp–40,000 bp).

#### Amplicon library preparation

All amplicon libraries were prepared using fusion PCR based on two nuclear ribosomal target sequences, internal transcribed spacers nrITS1 and nrITS2. PGM fusion primers were based on 17SE and 5.8 I1, and 5.8 I2 and 26 SE, respectively^59^. The forward primers were labeled with unique 10 bp multiplex identifier (MID) tags and the reverse primers with uniform truncated P1 (trP1) tags. Thermal cycling was carried out in 25 μl reaction volumes, and each reaction contained 5 μl 5X Q5 reaction buffer (New England Biolabs Inc, UK), 1.5 μl 10 μM of each primer (Biolegio, the Netherlands), 0.5 μl 10 mM dNTPs, 0.25 μl 20 U/μl Q5 High-Fidelity DNA Polymerase (New England Biolabs Inc, UK), 5 μl 5X Q5 High GC enhancer, 10.75 μl of Milli-Q ultrapure water and 0.5 μl of template DNA. The following thermocycling protocol was used: 30 s of initial denaturation at 98 °C, followed by 35 cycles of denaturation at 98 °C for 10 s, annealing at 30 s, and elongation at 72 °C for 30 s, followed by a final elongation step at 72 °C for 2 min. The annealing temperature was 56 °C for nrITS1, and 71 °C for nrITS2.

#### Equimolar pool preparation

The size, purity and the molar concentration (nmol/l) of each amplicon library was measured using a Fragment Analyzer™ (Advanced Analytical Technologies, Inc., USA) and a DNF-910 dsDNA Reagent Kit (35 bp–1,500 bp). An equimolar pool (2 ng/μl/library) was prepared from the amplicon libraries using the Biomek 4000 Laboratory Automation Workstation (Beckman Coulter, USA). Agencourt AMPure XP (Beckman Coulter, USA) was used for removal of unincorporated primers and nucleotides using the manufacturer’s instructions (Agencourt AMPure XP v. B37419AA). The total concentration of the purified pooled amplicon library stock and three serial dilutions (undiluted, 1/5, 1/10) were analyzed using the Fragment Analyzer™ (Advanced Analytical Technologies, Inc., USA) and DNF-488 High Sensitivity Genomic DNA Analysis Kit in order to identify the optimum concentration range for the template preparation.

#### High throughput sequencing

An Ion Chef (Life Technologies (LT), Thermo-Fisher Scientific, USA) was used to prepare pooled Ion AmpliSeq libraries (LT) for emulsion PCR and to load the sequencing chips. The input DNA template concentration was adjusted to the number of Ion Sphere Particles (ISPs) and added to the emulsion PCR master mix. The emulsion PCR was done using the Ion Chef, and template-positive ISPs were enriched and loaded on an Ion 318 v2 Chip (LT) and sequenced on an Ion Torrent Personal Genome Machine (LT) using an Ion PGM Sequencing 400 kit (LT). Sequencing read data was analyzed and demultiplexed into FASTQ files per sample using Torrent Suite version 5.0.4 (LT).

#### Bioinformatics analysis

FASTQ read files were processed using the HTS-barcode-checker pipeline^60^ available as a Galaxy pipeline at the Naturalis Biodiversity Center (http://145.136.240.164:8080/). Using the HTS pipeline, nrITS1 and nrITS2 primer sequences were used to demultiplex the sequencing reads per sample and to filter out reads that did not match any of the primers. PRINSEQ^61^ was used to determine filtering and trimming values based on read lengths and Phred read quality^62^. All reads with a mean Phred quality score of less than 26 were filtered out, as well as reads with a length of less than 300 bp. Remaining reads were trimmed to a maximum length of 440 bp for nrITS1 and 350 bp for nrITS2. CD-HIT-EST^63^ was used to cluster reads into molecular operational taxonomic units (MOTUs) defined by a sequence similarity of >99% and a minimum number of ten reads. The consensus sequences of non-singleton MOTUs were queried using BLAST^64^ against a reference nucleotide sequence database, with a maximum e-value of 0.05, a minimum hit length of 100 bp and sequence identity of >97%. The number of reads per MOTU, as well as the BLAST results per MOTU, were compiled using custom scripts from the HTS Barcode Checker pipeline^60^. The reference sequence database consisted of a local copy of the NCBI/GenBank nucleotide database.

### Data availability

Ion-Torrent amplicon read data is deposited in DRYAD: doi:10.5061/dryad.32j7r.

## Electronic supplementary material

Supplementary Data

Supplementary Data S7
