# Supplementary material for: Comparative authentication of Hypericum perforatum herbal products using DNA metabarcoding, TLC and HPLC-MS
Source: Sci Rep. 2017 May 2;7:1291. doi: 10.1038/s41598-017-01389-w (PMC5431008; doi:10.1038/s41598-017-01389-w)
Supplement: Supplementary file 1 — Supplementary Data [file 41598_2017_1389_MOESM1_ESM.pdf]

Comparative authentication of Hypericum perforatum herbal products using DNA metabarcoding, TLC and HPLC-MS

Ancuta Cristina Raclariu, Ramona Paltinean, Laurian Vlase, Aurélie Labarre, Vincent Manzanilla, Mihael Cristin Ichim, Gianina Crisan, Anne Krag Brysting, Hugo de Boer

Supplementary Information

Supplementary Figure S1. TLC chromatograms

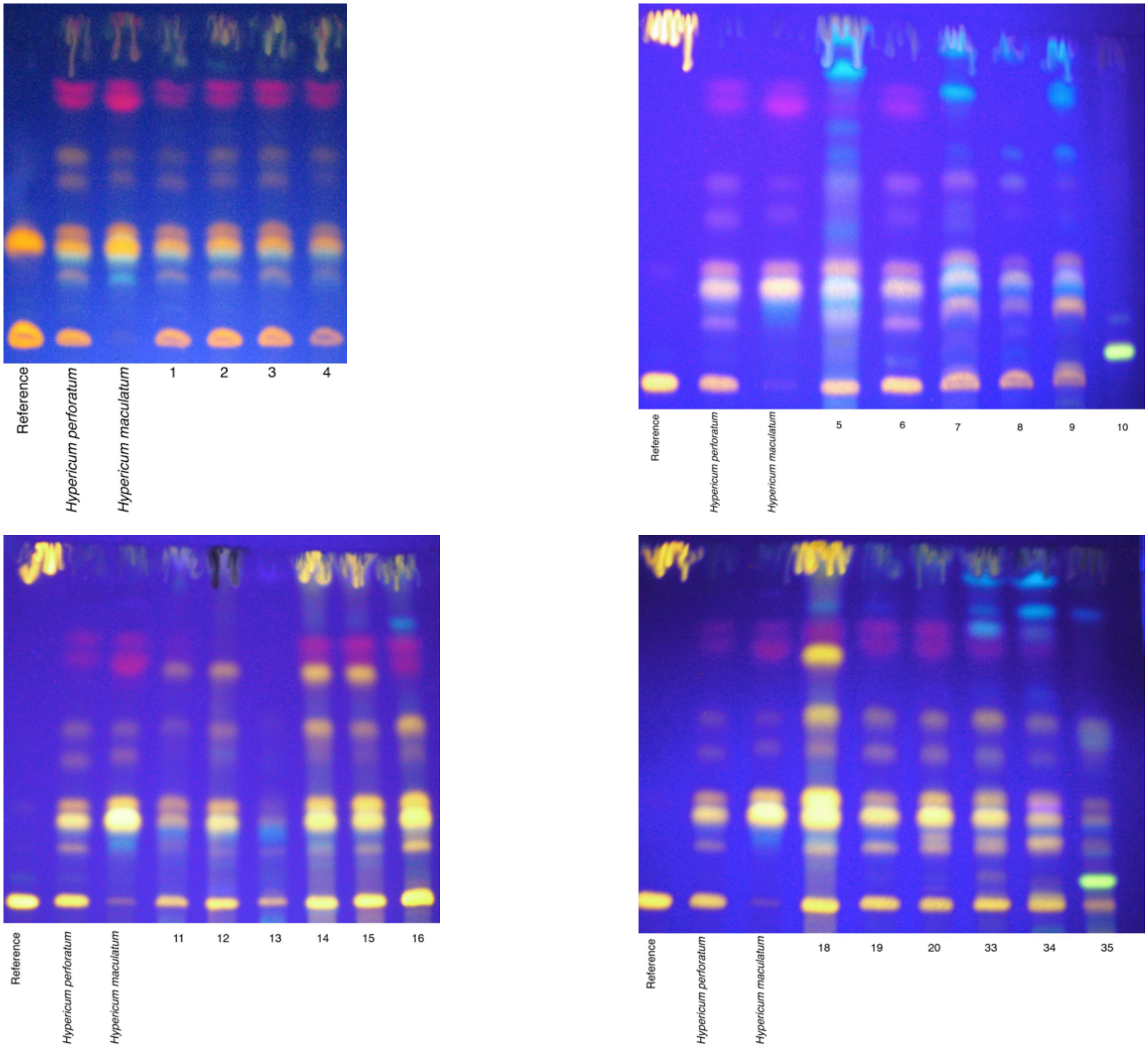

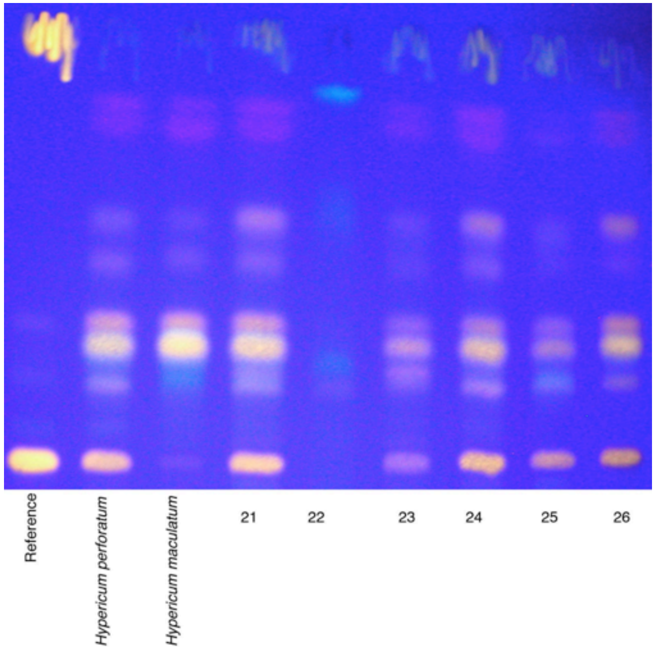
Reference
Hypericum perforatum
Hypericum maculatum
21
22
23
24
25
26

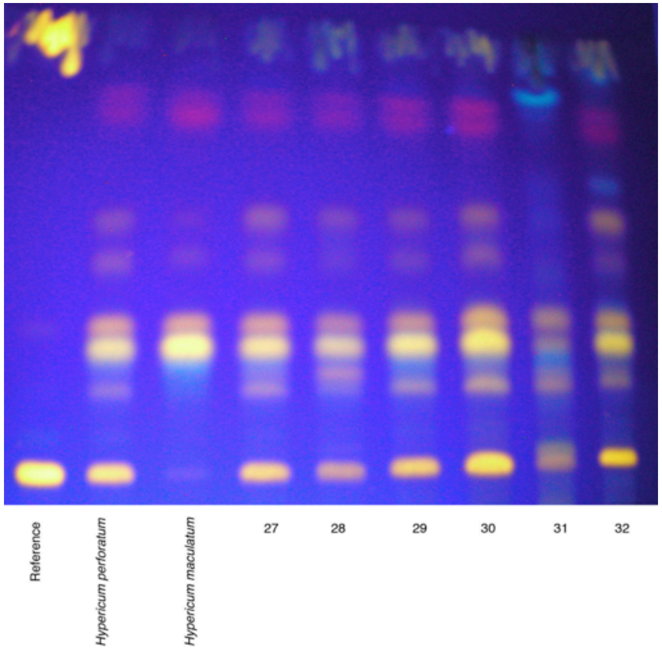
Reference
Hypericum perforatum
Hypericum maculatum
27
28
29
30
31
32

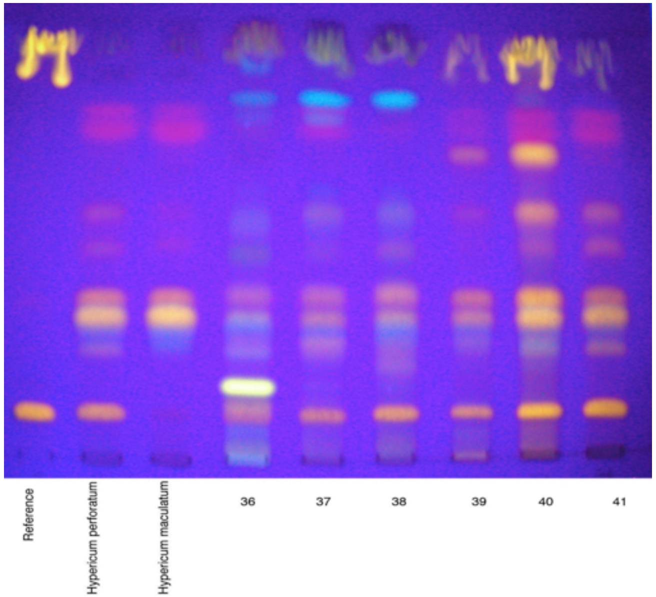
Reference
Hypericum perforatum
Hypericum maculatum
36
37
38
39
40
41

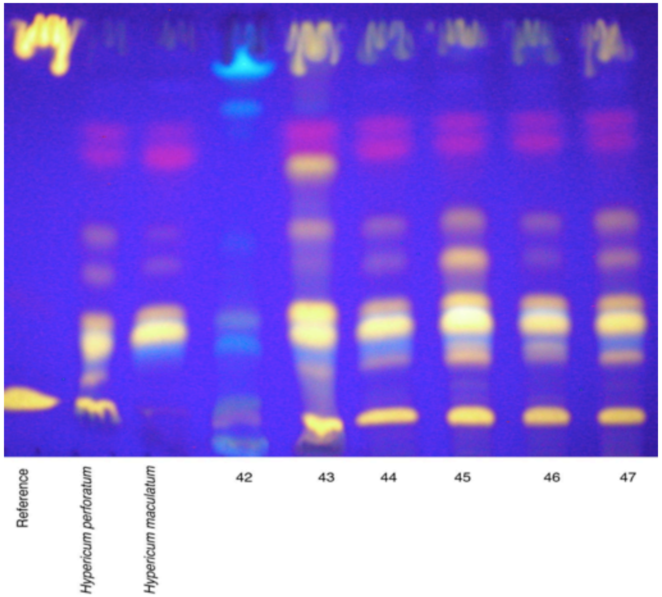
Reference
Hypericum perforatum
Hypericum maculatum
42
43
44
45
46
47

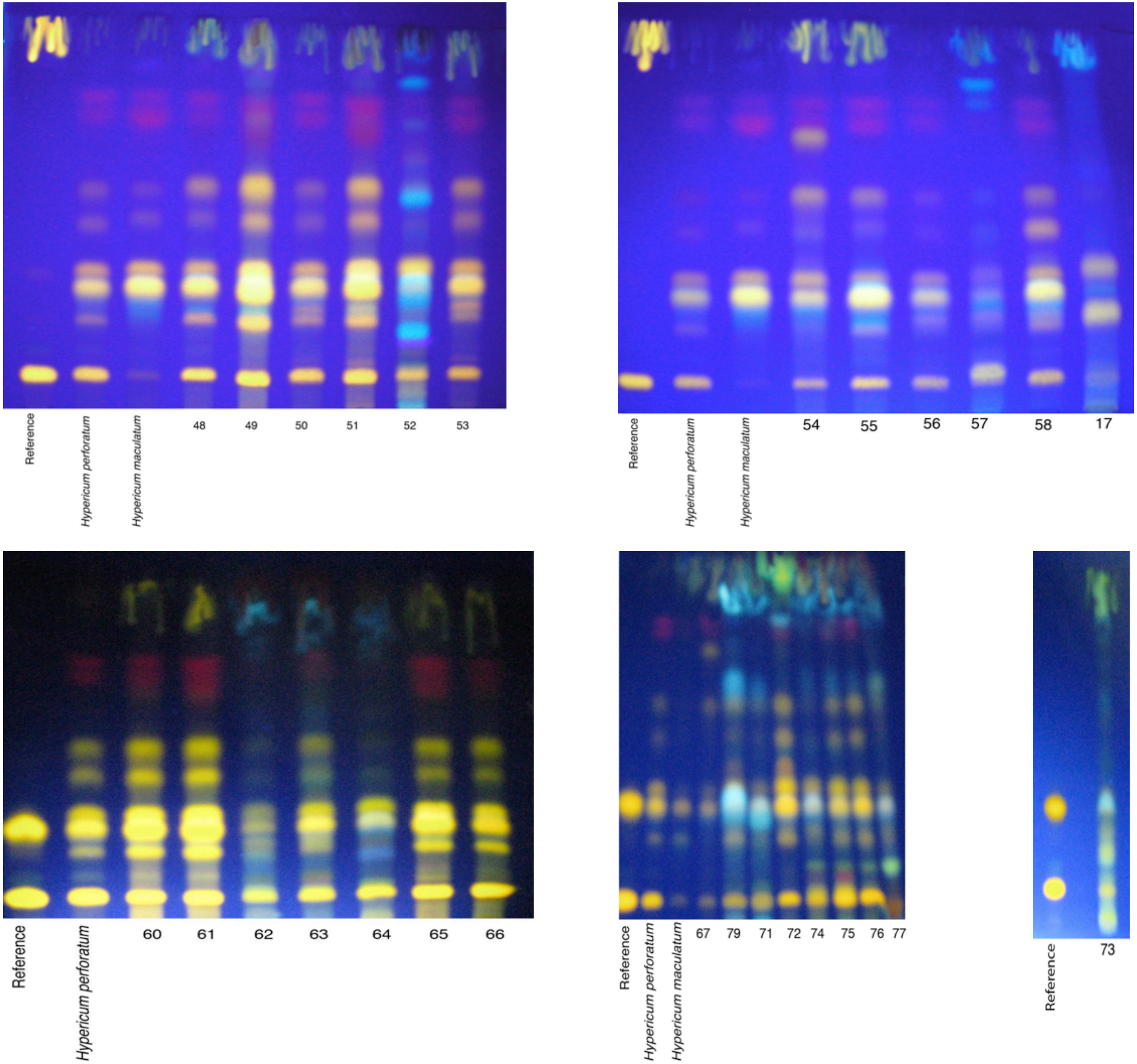
Reference
Hypericum perforatum
Hypericum maculatum
48
49
50
51
52
53
Reference
Hypericum perforatum
Hypericum maculatum
54
55
56
57
58
17
Reference
Hypericum perforatum
60
61
62
63
64
65
66
Reference
Hypericum perforatum
Hypericum maculatum
67
79
71
72
74
75
76
77
Reference
73

Supplementary Table S2. TLC results

Sample no.	Product type	Rutin	Hyperoside	Hypericin	Pseudohypericin	*Hypericum perforatum*	Other *Hypericum species*	No *Hypericum* detected	Extraction failed
1	Herbal tea	+	+	+	+	+			
2	Herbal tea	+	+	+	+	+			
3	Herbal tea	+	+	+	+	+			
4	Herbal tea	+	+	+	+	+			
5	Herbal tea	+	+	±	+	+			
6	Capsules	+	+	+	+	+			
7	Capsules	+	+	-	-		+		
8	Capsules	+	+	-	-		+		
9	Capsules	+	+	-	-		+		
10	Tablets	-	-	-	-			+	
11	Tablets	+	+	+	+	+			
12	Tablets	+	+	-	-		+		
13	Capsules	-	-	-	-			+	
14	Capsules	+	+	+	+	+			
15	Capsules	+	+	+	+	+			
16	Tablets	+	+	+	+	+			
17	Herbal tea	-	-	-	-			+	
18	Tablets	+	+	+	+	+			
19	Herbal tea	+	+	+	+	+			
20	Herbal tea	+	+	+	+	+			
21	Herbal tea	+	+	+	+	+			
22	Herbal tea	-	-	-	-			+	
23	Herbal tea	+	+	+	+	+			
24	Herbal tea	+	+	+	+	+			
25	Herbal tea	+	+	±	±	+			
26	Herbal tea	+	+	+	+	+			
27	Herbal tea	+	+	+	+	+			
28	Herbal tea	+	+	+	+	+			
29	Herbal tea	+	+	+	+	+			
30	Herbal tea	+	+	+	+	+			
31	Herbal tea	+	+	-	±		+		
32	Herbal tea	+	+	+	+	+			
33	Herbal tea	+	+	±	+	+			
34	Herbal tea	+	+	±	+	+			
35	Herbal tea	+	+	±	±		+		
36	Herbal tea	+	±	±	±		+		
37	Herbal tea	+	±	±	+	+			
38	Herbal tea	+	±	+	±	+			
39	Tablets	+	±	+	+	+			
40	Tablets	+	+	+	+	+			
41	Capsules	+	+	+	+	+			
42	Capsules	+	-	-	-			+	
43	Capsules	+	+	+	+	+			
44	Herbal tea	+	+	+	+	+			
45	Herbal tea	+	+	+	+	+			
46	Herbal tea	+	+	+	+	+			
47	Capsules	+	+	+	+	+			
48	Tablets	+	+	+	+	+			
49	Tablets	+	+	+	+	+			
50	Herbal tea	+	+	+	+	+			
51	Capsules	+	+	+	+	+			
52	Herbal tea	+	±	+	±	+			
53	Tablets	+	+	+	+	+			
54	Capsules	+	+	+	+	+			
55	Herbal tea	+	+	+	+	+			
56	Herbal tea	+	+	+	+	+			
57	Herbal tea	+	±	-	±	+			
58	Herbal tea	+	+	+	+	+			
59	Extract								+
60	Tablets	+	+	+	+	+			
61	Tablets	+	+	+	+	+			
62	Herbal tea	+	+	-	-		+		
63	Herbal tea	+	+	±	±	+			
64	Herbal tea	+	+	±	±		+		
65	Tablets	+	+	+	+	+			
66	Capsules	+	+	+	+	+			
67	Tablets	+	+	+	+	+			
68	Extract								+
69	Extract								+
70	Herbal tea	+	-	-	-			+	
71	Herbal tea	+	-	-	-			+	
72	Capsules	+	+	±	±	+			
73	Extract	+	-	-	-			+	
74	Herbal tea	+	-	-	-			+	
75	Herbal tea	+	+	±	±	+			
76	Herbal tea	+	+	+	+	+			
77	Herbal tea	-	-	-	-			+	
78	Extract								+

Supplementary Table S3. Hyperforin and hypericin concentrations measured in different species of *Hypericum* in µg/g dry weight

**Species**	**Hyperforin (µg/g)**	**Hypericin (µg/g)**	**Source**
H. androsaemum L.	90	0	Smelcerovic et al. 2008
H. aviculariifolium Jaub.& Spach	20	660	Smelcerovic et al. 2008
H. barbatum Jacq.	70	300	Smelcerovic et al. 2006
H. barbatum Jacq.	70	660	Smelcerovic and Spiteller, 2006
**Average** *H. barbatum*	70	300-660	
H. bithynicum Boiss.	150	1050	Smelcerovic et al. 2008
H. elegans Stephan ex Willd.	82.41	29	This study
H. heterophyllum Vent.	80	510	Smelcerovic et al. 2008
H. hirsutum L.	60	40	Smelcerovic et al. 2006
H. hirsutum L.	50	250	Smelcerovic and Spiteller, 2006
H. hirsutum L. flowers	0	2020	Umek et al. 1999
H. hirsutum L. herb	0	440	Umek et al. 1999
H. hirsutum L.	200	540	Smelcerovic et al. 2008
**Average** *H. hirsutum*	0-540	40-2020	
H. humifusum L. flowers	0	1640	Umek et al. 1999
H. humifusum L. herb	0	1180	Umek et al. 1999
**Average** *H. humifusum*	0	117-1640	
H. hyssopifolium Vill.	40	520	Smelcerovic et al. 2008
H. linarioides Bosse	20	20	Smelcerovic et al. 2006
H. linarioides Bosse	0	40	Smelcerovic and Spiteller, 2006
H. linarioides Bosse	0	340	Smelcerovic et al. 2008
**Average** *H. linarioides*	0-349	20-40	
H. maculatum Crantz	50	30	Smelcerovic et al. 2006
H. maculatum Crantz (RO)	0	142	This study
H. maculatum Crantz	180	70	Smelcerovic and Spiteller, 2006
H. maculatum Crantz (NO)	35	46	This study
H. maculatum Crantz flowers	0	1870	Umek et al. 1999
H. maculatum Crantz herb	0	460	Umek et al. 1999
**Average** *H. maculatum*	0-180	30-1870	
H. montbretii Spach	3450	740	Smelcerovic et al. 2008
H. montanum L.	0	1130	Smelcerovic et al. 2008
H. montanum L. herb	0	450	Umek et al. 1999
**Average** *H. montanum*	0-1130	0-450	
H. nummularioides Trautv.	250	200	Smelcerovic et al. 2008
H. olympicum L.	3955	11	This study
H. olympicum L.	20	50	Smelcerovic and Spiteller, 2006
**Average** *H. olympicum*	20-3955	11-50	
H. origanifolium Willd.	0	0	Smelcerovic et al. 2008
H. patulum Thunb.	8532	153	This study
H. perfoliatum L.	140	290	Smelcerovic et al. 2008
H. perforatum L.	4925	151	This study
H. perforatum L.	3550	170	Smelcerovic and Spiteller, 2006
H. perforatum L. flowers (2)	13590	5200	Umek et al. 1999
H. perforatum L. herb (1)	6010	1690	Umek et al. 1999
H. perforatum L.	5460	3470	Smelcerovic et al. 2008
**Average** *H. perforatum*	3470-13590	151-5200	
H. orientale L.	30	20	Smelcerovic et al. 2008
H. polyphyllum Boiss. & Balansa	6030	18	This study
H. pruinatum Boiss.& Balansa	50	360	Smelcerovic et al. 2008
H. richeri Vill.	360	450	Smelcerovic and Spiteller, 2006
H. rumeliacum Boiss.	70	180	Smelcerovic et al. 2006
H. rumeliacum Boiss.	150	230	Smelcerovic and Spiteller, 2006
**Average** *H. rumeliacum*	70-150	180-230	
H. scabrum L.	20	40	Smelcerovic et al. 2008
H. tetrapterum Fr.	110	90	Smelcerovic et al. 2006
H. tetrapterum Fr.	270	150	Smelcerovic and Spiteller, 2006
H. tetrapterum Fr. flowers	0	2230	Umek et al. 1999
H. tetrapterum Fr. herb	0	1100	Umek et al. 1999
**Average** *H. tetrapterum*	0-270	90-2230	
H. triquetrifolium Turra	50	4560	Smelcerovic et al. 2008

(1) An average of 22 samples. (2) An average of 21 samples.

**Supplementary Table S4**. HPLC-MS results								
**Sample no.**	**Product type**	**Hyperforin (µg/g of herbal product)**	**Hypericin (µg/g of herbal product)**	**Total hypericins (%)**	**Hyperforin absent, but hypericin present (=Hypericum sp.)**	**Hyperforin and hypericin present (=Hypericum sp.)**	**Extraction failed**	**Hyperforin larger than hypericin**
1	Herbal tea	444.09	53.14	0.005	0	1	0	1
2	Herbal tea	1333	81.25	0.008	0	1	0	1
3	Herbal tea	2491.83	109.96	0.011	0	1	0	1
4	Herbal tea	1398.09	88.1	0.009	0	1	0	1
5	Herbal tea	109.41	6.59	0.001	0	1	0	1
6	Capsules	1980.92	56.13	0.006	0	1	0	1
7	Capsules	2.79	1.8	0.000	0	1	0	1
8	Capsules	3.65	0	0.000	0	0	0	1
9	Capsules	7	1.3	0.000	0	1	0	1
10	Tablets	16.86	0	0.000	0	0	0	1
11	Tablets	17	4.12	0.000	0	1	0	1
12	Tablets	104.18	1.366	0.000	0	1	0	1
13	Capsules	0	0	0.000	2	0	0	0
14	Capsules	4005.73	287.26	0.029	0	1	0	1
15	Capsules	2421.38	126.15	0.013	0	1	0	1
16	Tablets	580.35	40.82	0.004	0	1	0	1
17	Herbal tea	4.79	0	0.000	0	0	0	1
18	Tablets	342.41	164.82	0.016	0	1	0	1
19	Herbal tea	3868.03	87.95	0.009	0	1	0	1
20	Herbal tea	270.47	90.33	0.009	0	1	0	1
21	Herbal tea	1855.18	82.59	0.008	0	1	0	1
22	Herbal tea	0	0	0.000	2	0	0	0
23	Herbal tea	972.99	43.43	0.004	0	1	0	1
24	Herbal tea	4650.58	162.25	0.016	0	1	0	1
25	Herbal tea	1331.71	45.41	0.005	0	1	0	1
26	Herbal tea	6077.32	90.31	0.009	0	1	0	1
27	Herbal tea	6691.28	63.94	0.006	0	1	0	1
28	Herbal tea	1034.96	74.02	0.007	0	1	0	1
29	Herbal tea	5043.67	106.77	0.011	0	1	0	1
30	Herbal tea	1044.15	97.23	0.010	0	1	0	1
31	Herbal tea	198.07	1.78	0.000	0	1	0	1
32	Herbal tea	63.48	24.87	0.002	0	1	0	1
33	Herbal tea	46.86	27.48	0.003	0	1	0	1
34	Herbal tea	94.68	12.75	0.001	0	1	0	1
35	Herbal tea	83.75	1.71	0.000	0	1	0	1
36	Herbal tea	364.5	6.29	0.001	0	1	0	1
37	Herbal tea	165.38	9.29	0.001	0	1	0	1
38	Herbal tea	51.49	4.52	0.000	0	1	0	1
39	Tablets	17.57	5.23	0.001	0	1	0	1
40	Tablets	56.81	42.53	0.004	0	1	0	1
41	Capsules	0	61.47	0.006	1	0	0	0
42	Capsules	0	0	0.000	2	0	0	0
43	Capsules	674.21	85.15	0.009	0	1	0	1
44	Herbal tea	237.28	106.82	0.011	0	1	0	1
45	Herbal tea	98.81	86.4	0.009	0	1	0	1
46	Herbal tea	31.09	109.02	0.011	0	1	0	0
47	Capsules	0	129.84	0.013	1	0	0	0
48	Tablets	0	13.25	0.001	1	0	0	0
49	Tablets	428.1	53.07	0.005	0	1	0	1
50	Herbal tea	80.48	118.53	0.012	0	1	0	0
51	Capsules	2797.5	298.34	0.030	0	1	0	1
52	Herbal tea	318.52	10.24	0.001	0	1	0	1
53	Tablets	116.69	96.4	0.010	0	1	0	1
54	Capsules	758.06	68.13	0.007	0	1	0	1
55	Herbal tea	0	80.23	0.008	1	0	0	0
56	Herbal tea	1108.58	79.29	0.008	0	1	0	1
57	Herbal tea	80.61	12.3	0.001	0	1	0	1
58	Herbal tea	4925.43	151.97	0.015	0	1	0	1
59	Extract			0.000	0	0	1	0
60	Tablets	31.03	42.61	0.004	0	1	0	0
61	Tablets	89.71	85.74	0.009	0	1	0	1
62	Herbal tea	72	3.58	0.000	0	1	0	1
63	Herbal tea	219.68	15.02	0.002	0	1	0	1
64	Herbal tea	84.6	1.99	0.000	0	1	0	1
65	Tablets	683.63	109.75	0.011	0	1	0	1
66	Capsules	2713.27	92.4	0.009	0	1	0	1
67	Tablets	52	24.78	0.002	0	1	0	1
68	Extract			0.000	0	0	1	0
69	Extract			0.000	0	0	1	0
70	Herbal tea	0	1.83	0.000	1	0	0	0
71	Herbal tea	51.21	1.39	0.000	0	1	0	1
72	Capsules	19.03	6.33	0.001	0	1	0	1
73	Extract	0	0	0.000	2	0	0	0
74	Herbal tea	44.48	2.39	0.000	0	1	0	1
75	Herbal tea	147.92	8.8	0.001	0	1	0	1
76	Herbal tea	689.36	14.66	0.001	0	1	0	1
77	Herbal tea	223	2.43	0.000	0	1	0	1
78	Extract			0.000	0	0	1	0

Supplementary Table S5. HTS success

Product	Reads		nrITS1			nrITS2		
No.	No. of bases before demultiplexing	No. of reads before demultiplexing	Amplicon concentration (ng/ul)	No. of reads	No. of bases (>Q20)	Amplicon concentration (ng/ul)	No. of reads	No. of bases (>Q20)
1	62722750	285184	3.70	258020	19830698	4.69	16022	1578942
2	47358	307	4.10	6	514	0.95	238	12464
3	521886	2161	2.91	652	58148	2.88	1361	118469
4	47455333	208055	3.30	115965	9196791	1.68	79050	7101592
5	22231491	145827	2.96	44775	3356973	1.46	80022	3183064
6	12558328	63808	4.02	21329	1564485	1.42	35834	2711535
7	17804	64	1.72	24	2925	1.12	32	2779
8	1386042	5492	0.39	3331	381846	0.25	1724	103955
9	8988	55	1.55	27	1662	0.89	26	1065
10	75383619	278105	6.06	178262	20295668	4.33	83862	0
11	2370	8	0.89	8	891	0.88	0	0
12	35056989	151616	0.15	0	0	0.13	0	0
13	4389	14	0.17	10	1424	0.13	0	0
14	0	0	0.12	0	0	0.16	0	0
15	27614658	86515	1.18	68820	8868686	0.91	15439	1711591
16	0	0	0.25	0	0	0.13	0	0
17	1544147	5029	0.06	3140	336506	0.21	734	86450
18	1903	10	0.12	9	599	0.30	0	0
19	10037	34	1.43	29	2997	2.59	4	513
20	5212	23	2.66	18	1582	4.27	3	9
21	138559130	566977	2.14	205015	19729261	3.50	320385	30508847
22	2028	8	6.84	0	0	1.86	3	293
23	1402	4	8.51	2	272	4.08	2	291
24	83221168	319287	4.71	121339	11588669	1.16	181237	18954323
25	6077	21	1.39	16	1683	1.99	2	266
26	9830	35	4.25	27	2806	1.28	2	37
27	71059267	278718	4.53	138667	13045974	1.74	125391	12717263
28	6294	32	2.14	19	1868	0.69	7	77
29	56684931	226221	3.22	183332	17339018	3.68	29203	2452257
30	53107442	242925	2.00	192134	14919716	3.10	34767	3221126
31	1350	7	2.43	0	0	2.30	0	0
32	29831458	114441	4.57	64443	6636146	5.37	42496	4057305
33	3667	13	4.08	2	116	5.72	1	4
34	22082	95	1.70	71	7040	3.34	12	334
35	22344764	73322	5.61	44708	5422673	6.83	26203	3230857
36	6503	21	4.99	10	1283	2.92	8	838
37	98800823	441576	5.05	208647	19367123	2.14	192205	15149014
38	62730698	259246	4.09	165272	14664712	3.37	77438	7002425
39	43379160	45620	1.67	97249	11054541	1.26	43372	5214116
40	459	2	1.61	0	0	1.17	0	0
41	15171	69	2.62	0	0	1.12	2	303
42	80345560	299829	3.91	198256	21106677	4.52	80502	7903473
43	46950181	168677	4.36	92104	10485352	1.87	67209	6991979
44	164237026	672962	3.76	591566	54135759	4.11	48363	2761743
45	1282	4	9.87	2	244	0.23	2	237
46	102410827	376539	2.62	283073	29390005	2.54	78775	7969442
47	125	2	0.08	1	17	0.04	0	0
48	44196732	150787	0.18	141146	15649176	0.07	4419	299653
49	27291	100	0.64	69	7625	0.19	0	0
50	76087488	287856	7.65	105680	8446677	0.74	171362	19150153
51	25615264	87625	0.18	12048	1326009	0.14	18	8462273
52	4911	33	8.22	8	355	0.41	13	990
53	337	2	0.06	2	101	0.11	0	0
54	2913	13	4.43	0	0	2.05	13	941
55	27503996	94631	0.30	91606	9700289	0.18	103	2256
56	2545	10	2.59	4	339	1.41	4	586
57	21533	154	2.89	0	0	1.33	104	4579
58	8840	36	4.28	24	2557	0.36	6	439
59	5273773	22446	0.20	6584	566608	0.17	15042	1226366
60	1235	6	0.19	1	1	0.39	3	254
61	29752850	118302	0.22	74241	7862582	0.50	39185	2473510
62	8383	28	9.32	6	722	0.39	21	2455
63	5413	17	1.63	8	1154	0.35	1	1
64	7966	32	7.60	0	0	0.33	31	3036
65	17529659	52196	0.25	50670	6663535	0.49	261	28162
66	0	0	0.12	0	0	0.19	0	0
67	0	0	0.12	0	0	0.05	0	0
68	0	0	0.20	0	0	0.15	0	0
69	0	0	0.17	0	0	0.21	0	0
70	10179	41	1.97	29	2816	1.99	0	0
71	55062199	220984	1.45	70947	7534569	4.36	131918	12071501
72	114616133	435823	1.97	180484	21776940	0.29	220087	20829439
73	26683841	106119	2.30	17391	1481069	0.57	83635	8025957
74	265805068	1044831	1.58	303233	28037295	2.21	654831	70358140
75	66354277	298951	2.47	139169	12433535	1.75	131723	10373161
76	86083702	377066	1.82	217576	19943312	0.84	119966	10397432
77	150477835	596181	1.61	292909	29389441	1.40	253969	24631492
78	60778605	202803	0.92	120092	14223598	0.95	72348	8575923

Supplementary Information Figure S6

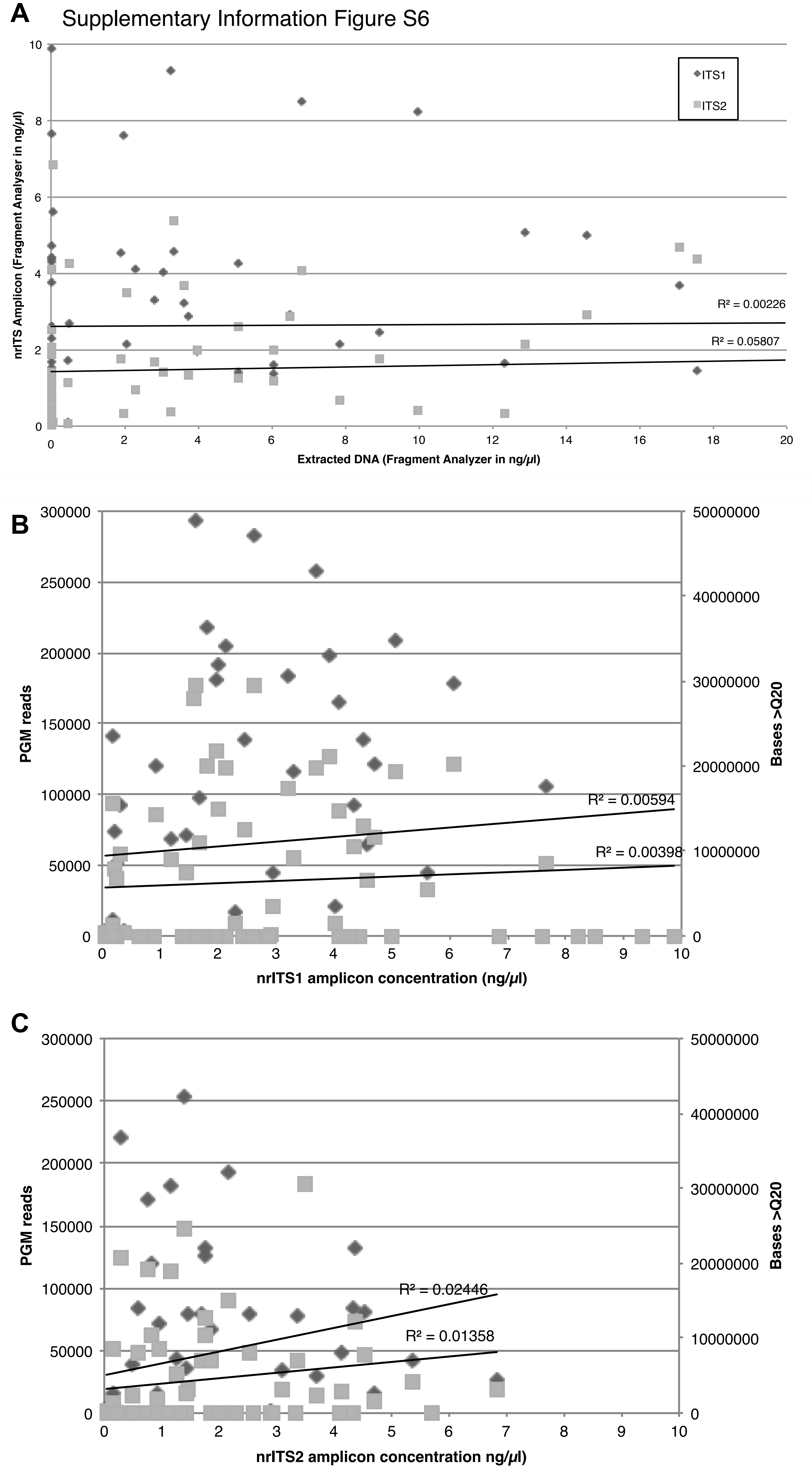

Supplementary Figure S8. nrITS1 heatmap of relative abundances of normalized read numbers

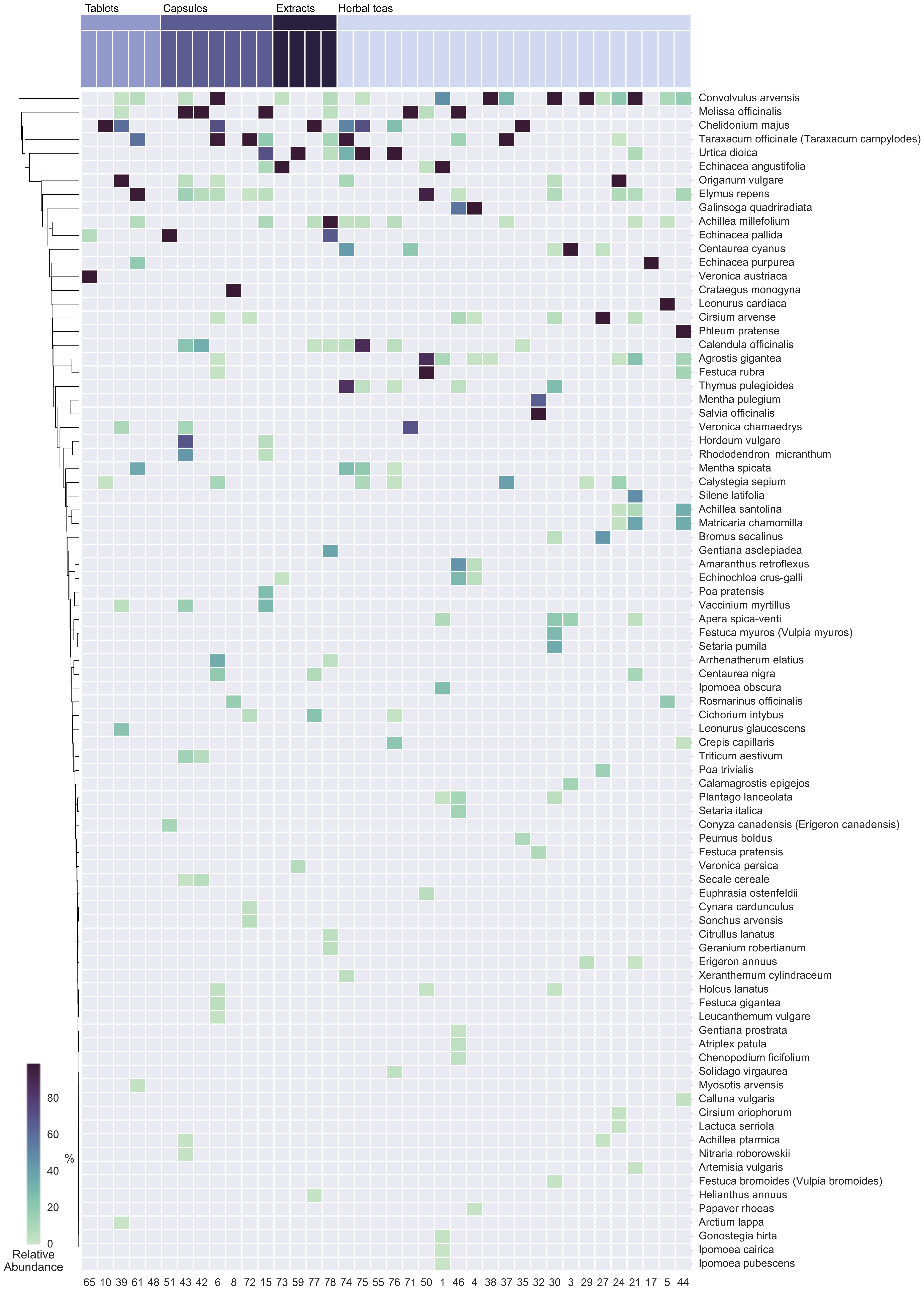

Supplementary Figure S9. nrITS2 heatmap of relative abundance of normalized read numbers

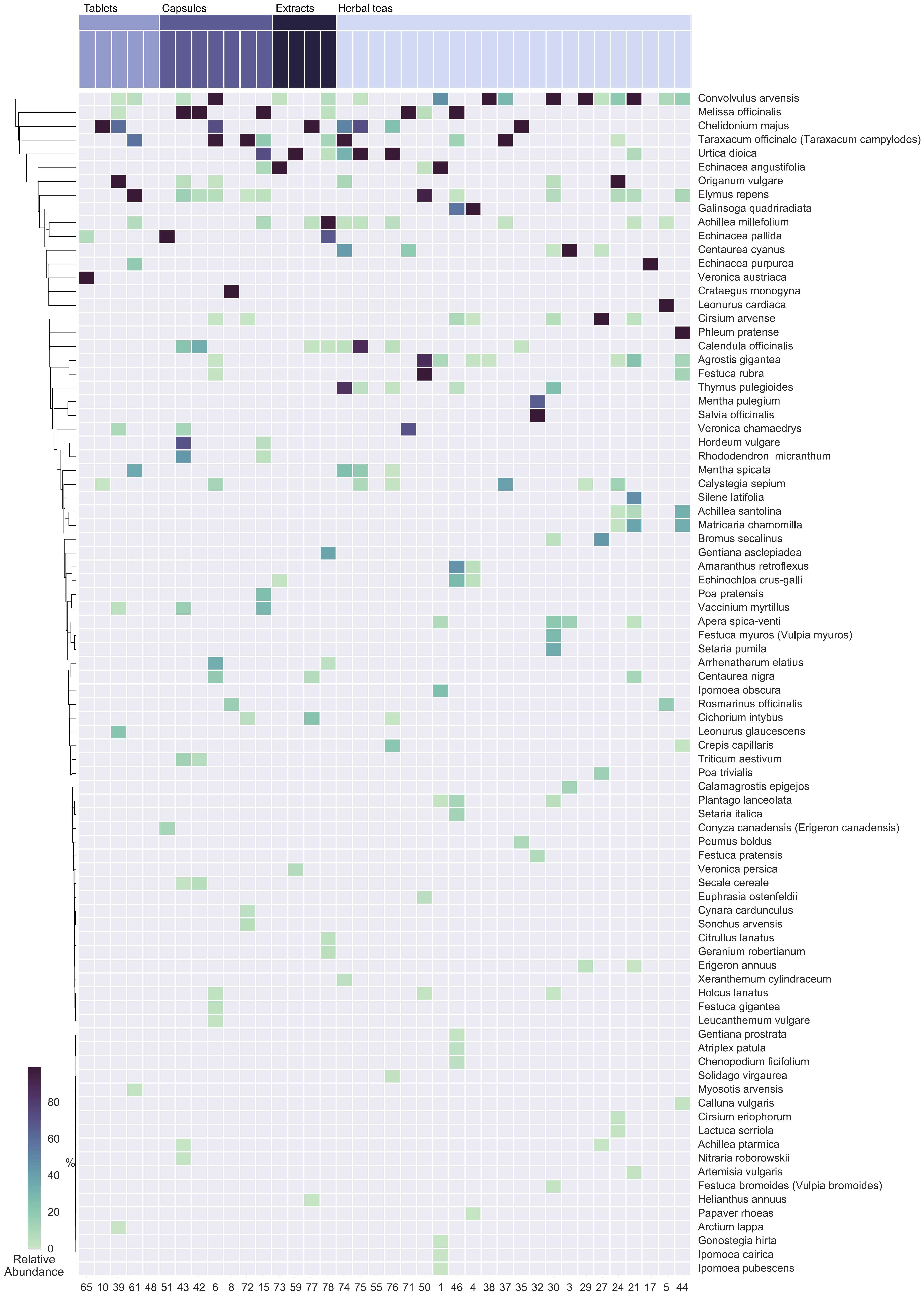

Supplementary Table S10. Information about products

Sample no.	Number of species on label	Scientific names of the plant ingredients	Product type	Country of origin	Country of acquisition	Vendor type	Product clasification
1	1	*Hypericum perforatum* L.	Herbal teas	Romania	Romania	Pharmacy	Unknown
2	1	*Hypericum perforatum* L.	Herbal teas	Romania	Romania	Pharmacy	Food supplement
3	1	*Hypericum perforatum* L.	Herbal teas	Romania	Romania	Pharmacy	Food supplement
4	1	*Hypericum perforatum* L.	Herbal teas	Romania	Romania	Pharmacy	Food supplement
5	10	*Achillea millefolium* L.	Herbal teas	Romania	Romania	Pharmacy	Food supplement
5	10	*Crataegus monogyna* Jacq.	Herbal teas	Romania	Romania	Pharmacy	Food supplement
5	10	*Equisetum arvense* L.	Herbal teas	Romania	Romania	Pharmacy	Food supplement
5	10	*Glycyrrhiza glabra* L.	Herbal teas	Romania	Romania	Pharmacy	Food supplement
5	10	*Hypericum perforatum* L.	Herbal teas	Romania	Romania	Pharmacy	Food supplement
5	10	*Lavandula angustifolia subsp. angustifolia (syn. Lavandula officinalis* Chaix)	Herbal teas	Romania	Romania	Pharmacy	Food supplement
5	10	*Leonurus cardiaca* L.	Herbal teas	Romania	Romania	Pharmacy	Food supplement
5	10	*Rosmarinus officinalis* L.	Herbal teas	Romania	Romania	Pharmacy	Food supplement
5	10	*Sambucus nigra* L.	Herbal teas	Romania	Romania	Pharmacy	Food supplement
5	10	*Valeriana officinalis* L.	Herbal teas	Romania	Romania	Pharmacy	Food supplement
6	2	*Hypericum perforatum* L.	Capsules	Romania	Romania	E-commerce	Food supplement
6	2	Panax ginseng	Capsules	Romania	Romania	E-commerce	Food supplement
7	7	*Arctium lappa* L.	Capsules	Romania	Romania	Pharmacy	Food supplement
7	7	*Elaeagnus rhamnoides* (L.) A.Nelson *(Hippophae rhamnoides* L.)	Capsules	Romania	Romania	Pharmacy	Food supplement
7	7	*Hypericum perforatum* L.	Capsules	Romania	Romania	Pharmacy	Food supplement
7	7	*Lycopodium clavatum* L.	Capsules	Romania	Romania	Pharmacy	Food supplement
7	7	*Salix alba* L.	Capsules	Romania	Romania	Pharmacy	Food supplement
7	7	*Taraxacum officinale* Webb	Capsules	Romania	Romania	Pharmacy	Food supplement
7	7	*Thymus serpyllum* L.	Capsules	Romania	Romania	Pharmacy	Food supplement
8	7	*Acorus calamus* var. americanus Raf.	Capsules	Romania	Romania	Pharmacy	Food supplement
8	7	*Allium ursinum* L.	Capsules	Romania	Romania	Pharmacy	Food supplement
8	7	*Elaeagnus rhamnoides* (L.) A.Nelson *(Hippophae rhamnoides* L.)	Capsules	Romania	Romania	Pharmacy	Food supplement
8	7	*Hypericum perforatum* L.	Capsules	Romania	Romania	Pharmacy	Food supplement
8	7	*Lycopodium clavatum* L.	Capsules	Romania	Romania	Pharmacy	Food supplement
8	7	*Mentha pulegium* L.	Capsules	Romania	Romania	Pharmacy	Food supplement
8	7	*Salvia officinalis* L.	Capsules	Romania	Romania	Pharmacy	Food supplement
9	9	*Achillea millefolium* L.	Capsules	Romania	Romania	Pharmacy	Food supplement
9	9	*Acorus calamus* var. americanus Raf.	Capsules	Romania	Romania	Pharmacy	Food supplement
9	9	*Calendula officinalis* L.	Capsules	Romania	Romania	Pharmacy	Food supplement
9	9	*Foeniculum vulgare* Mill.	Capsules	Romania	Romania	Pharmacy	Food supplement
9	9	*Hypericum perforatum* L.	Capsules	Romania	Romania	Pharmacy	Food supplement
9	9	*Mentha × piperita* L.	Capsules	Romania	Romania	Pharmacy	Food supplement
9	9	*Robinia pseudoacacia* L.	Capsules	Romania	Romania	Pharmacy	Food supplement
9	9	*Rubus idaeus* L.	Capsules	Romania	Romania	Pharmacy	Food supplement
9	9	*Taraxacum officinale* Webb	Capsules	Romania	Romania	Pharmacy	Food supplement
10	6	*Chelidonium majus* L.	Tablets	Romania	Romania	Pharmacy	Food supplement
10	6	*Cynara scolymus* L.	Tablets	Romania	Romania	Pharmacy	Food supplement
10	6	*Epilobium hirsutum* L.	Tablets	Romania	Romania	Pharmacy	Food supplement
10	6	*Humulus lupulus* L.	Tablets	Romania	Romania	Pharmacy	Food supplement
10	6	*Humulus lupulus* L.	Tablets	Romania	Romania	Pharmacy	Food supplement
10	6	*Hypericum perforatum* L.	Tablets	Romania	Romania	Pharmacy	Food supplement
10	6	*Valeriana officinalis* L.	Tablets	Romania	Romania	Pharmacy	Food supplement
11	5	*Calendula officinalis* L.	Tablets	Romania	Romania	Pharmacy	Food supplement
11	5	*Foeniculum vulgare* Mill.	Tablets	Romania	Romania	Pharmacy	Food supplement
11	5	*Hypericum perforatum* L.	Tablets	Romania	Romania	Pharmacy	Food supplement
11	5	*Origanum vulgare* L.	Tablets	Romania	Romania	Pharmacy	Food supplement
11	5	*Plantago* spp.	Tablets	Romania	Romania	Pharmacy	Food supplement
12	2	*Hypericum perforatum* L.	Tablets	Romania	Romania	Pharmacy	Food supplement
12	2	*Spirulina platensis* (Gomont) Geitler	Tablets	Romania	Romania	Pharmacy	Food supplement
13	5	*Humulus lupulus* L.	Capsules	Romania	Romania	Pharmacy	Food supplement
13	5	*Hypericum perforatum* L.	Capsules	Romania	Romania	Pharmacy	Food supplement
13	5	*Leonurus cardiaca* L.	Capsules	Romania	Romania	Pharmacy	Food supplement
13	5	*Tilia cordata* Mill.	Capsules	Romania	Romania	Pharmacy	Food supplement
13	5	*Valeriana officinalis* L.	Capsules	Romania	Romania	Pharmacy	Food supplement
14	2	*Griffonia simplicifolia* (DC.) Baill.	Capsules	China	Romania	Pharmacy	Food supplement
14	2	*Hypericum perforatum* L.	Capsules	China	Romania	Pharmacy	Food supplement
15	1	*Hypericum perforatum* L.	Capsules	Romania	Romania	Pharmacy	Unknown
16	1	*Hypericum perforatum* L.	Tablets	Romania	Romania	Pharmacy	Food supplement
17	5	*Foeniculum vulgare* Mill.	Herbal teas	Romania	Romania	Pharmacy	Food supplement
17	5	*Hypericum perforatum* L.	Herbal teas	Romania	Romania	Pharmacy	Food supplement
17	5	*Medicago sativa* L.	Herbal teas	Romania	Romania	Pharmacy	Food supplement
17	5	*Origanum vulgare* L.	Herbal teas	Romania	Romania	Pharmacy	Food supplement
17	5	*Salvia sclarea* L.	Herbal teas	Romania	Romania	Pharmacy	Food supplement
18	1	*Hypericum perforatum* L.	Tablets	USA	Romania	Pharmacy	Food supplement
19	1	*Hypericum perforatum* L.	Herbal teas	Romania	Romania	Pharmacy	Food supplement
20	1	*Hypericum perforatum* L.	Herbal teas	Poland	Romania	E-commerce	Food supplement
21	1	*Hypericum perforatum* L.	Herbal teas	Romania	Romania	Pharmacy	Food supplement
22	1	*Hypericum perforatum* L.	Herbal teas	Romania	Romania	E-commerce	Food supplement
23	1	*Hypericum perforatum* L.	Herbal teas	Poland	Romania	Supermarket	Unknown
24	1	*Hypericum perforatum* L.	Herbal teas	Romania	Romania	Pharmacy	Unknown
25	1	*Hypericum perforatum* L.	Herbal teas	Romania	Romania	Supermarket	Unknown
26	1	*Hypericum perforatum* L.	Herbal teas	Romania	Romania	Pharmacy	Unknown
27	1	*Hypericum perforatum* L.	Herbal teas	Romania	Romania	Pharmacy	Food supplement
28	1	*Hypericum perforatum* L.	Herbal teas	Romania	Romania	Pharmacy	Food supplement
29	1	*Hypericum perforatum* L.	Herbal teas	Romania	Romania	E-commerce	Food supplement
30	1	*Hypericum perforatum* L.	Herbal teas	Unknown	Romania	E-commerce	Food supplement
31	14	*Achillea millefolium* L.	Herbal teas	Romania	Romania	Health shop	Food supplement
31	14	*Alchemilla xanthochlora* Rothm. (syn. *Alchemilla vulgaris* L.)	Herbal teas	Romania	Romania	Health shop	Food supplement
31	14	*Calendula officinalis* L.	Herbal teas	Romania	Romania	Health shop	Food supplement
31	14	*Capsella bursa-pastoris* (L.) Medik.	Herbal teas	Romania	Romania	Health shop	Food supplement
31	14	*Cichorium intybus* L.	Herbal teas	Romania	Romania	Health shop	Food supplement
31	14	*Equisetum arvense* L.	Herbal teas	Romania	Romania	Health shop	Food supplement
31	14	*Galium verum* L.	Herbal teas	Romania	Romania	Health shop	Food supplement
31	14	*Geranium robertianum* L.	Herbal teas	Romania	Romania	Health shop	Food supplement
31	14	*Hypericum perforatum* L.	Herbal teas	Romania	Romania	Health shop	Food supplement
31	14	*Lamium album* L.	Herbal teas	Romania	Romania	Health shop	Food supplement
31	14	*Leonurus cardiaca* L.	Herbal teas	Romania	Romania	Health shop	Food supplement
31	14	*Matricaria chamomilla* L.	Herbal teas	Romania	Romania	Health shop	Food supplement
31	14	*Salvia officinalis* L.	Herbal teas	Romania	Romania	Health shop	Food supplement
31	14	*Thymus serpyllum* L.	Herbal teas	Romania	Romania	Health shop	Food supplement
32	7	*Acorus calamus* var. americanus Raf.	Herbal teas	Romania	Romania	E-commerce	Food supplement

32	7	*Allium ursinum* L.	Herbal teas	Romania	Romania	E-commerce	Food supplement
32	7	*Elaeagnus rhamnoides (L.)* A.Nelson (*Hippophae rhamnoides* L.)	Herbal teas	Romania	Romania	E-commerce	Food supplement
32	7	*Hypericum perforatum* L.	Herbal teas	Romania	Romania	E-commerce	Food supplement
32	7	*Lycopodium clavatum* L.	Herbal teas	Romania	Romania	E-commerce	Food supplement
32	7	*Mentha pulegium* L.	Herbal teas	Romania	Romania	E-commerce	Food supplement
33	7	*Salvia officinalis* L.	Herbal teas	Romania	Romania	E-commerce	Food supplement
33	7	*Arctium lappa* L.	Herbal teas	Romania	Romania	E-commerce	Food supplement
33	7	*Elaeagnus rhamnoides (L.)* A.Nelson (*Hippophae rhamnoides* L.)	Herbal teas	Romania	Romania	E-commerce	Food supplement
33	7	*Hypericum perforatum* L.	Herbal teas	Romania	Romania	E-commerce	Food supplement
33	7	*Lycopodium clavatum* L.	Herbal teas	Romania	Romania	E-commerce	Food supplement
33	7	*Salix alba* L.	Herbal teas	Romania	Romania	E-commerce	Food supplement
33	7	*Taraxacum officinale* Webb	Herbal teas	Romania	Romania	E-commerce	Food supplement
33		*Thymus serpyllum* L.	Herbal teas	Romania	Romania	E-commerce	Food supplement
34	9	*Achillea millefolium* L.	Herbal teas	Romania	Romania	Pharmacy	Food supplement
34	9	*Calendula officinalis* L.	Herbal teas	Romania	Romania	Pharmacy	Food supplement
34	9	*Foeniculum vulgare* Mill.	Herbal teas	Romania	Romania	Pharmacy	Food supplement
34	9	*Hypericum perforatum* L.	Herbal teas	Romania	Romania	Pharmacy	Food supplement
34	9	*Mentha × piperita* L.	Herbal teas	Romania	Romania	Pharmacy	Food supplement
34	9	*Origanum vulgare* L.	Herbal teas	Romania	Romania	Pharmacy	Food supplement
34	9	*Robinia pseudoacacia* L.	Herbal teas	Romania	Romania	Pharmacy	Food supplement
34	9	*Taraxacum officinale* Webb	Herbal teas	Romania	Romania	Pharmacy	Food supplement
34	9	*Urtica dioica* L.	Herbal teas	Romania	Romania	Pharmacy	Food supplement
35	8	*Calendula officinalis* L.	Herbal teas	Romania	Romania	Health shop	Food supplement
35	8	*Chelidonium majus* L.	Herbal teas	Romania	Romania	Health shop	Food supplement
35	8	*Cichorium intybus* L.	Herbal teas	Romania	Romania	Health shop	Food supplement
35	8	*Hypericum perforatum* L.	Herbal teas	Romania	Romania	Health shop	Food supplement
35		*Melilotus officinalis* subsp. *alba* (Medik.) H.Ohashi & Tateishi	Herbal teas	Romania	Romania	Health shop	Food supplement
35	8	*Mentha × piperita* L.	Herbal teas	Romania	Romania	Health shop	Food supplement
35	8	*Peumus boldus* Molina	Herbal teas	Romania	Romania	Health shop	Food supplement
35	8	*Silybum marianum* (L.) Gaertn. (syn. *Carduus marianus* L.)	Herbal teas	Romania	Romania	Health shop	Food supplement
36	10	*Achillea millefolium* L.	Herbal teas	Romania	Romania	Pharmacy	Food supplement
36	10	*Agrimonia eupatoria* L.	Herbal teas	Romania	Romania	Pharmacy	Food supplement
36	10	*Chelidonium majus* L.	Herbal teas	Romania	Romania	Pharmacy	Food supplement
36	10	*Convolvulus arvensis* L.	Herbal teas	Romania	Romania	Pharmacy	Food supplement
36	10	*Frangula dodonei* Ard.(*Rhamnus frangula* L.)	Herbal teas	Romania	Romania	Pharmacy	Food supplement
36	10	*Hypericum perforatum* L.	Herbal teas	Romania	Romania	Pharmacy	Food supplement
36	10	*Mentha × piperita* L.	Herbal teas	Romania	Romania	Pharmacy	Food supplement
36	10	*Rosa canina* L.	Herbal teas	Romania	Romania	Pharmacy	Food supplement
36	10	*Taraxacum officinale* Webb	Herbal teas	Romania	Romania	Pharmacy	Food supplement
36	10	*Zea mays* L.	Herbal teas	Romania	Romania	Pharmacy	Food supplement
37	4	*Achillea millefolium* L.	Herbal teas	Romania	Romania	Pharmacy	Food supplement
37	4	*Convolvulus arvensis* L.	Herbal teas	Romania	Romania	Pharmacy	Food supplement
37	4	*Hypericum perforatum* L.	Herbal teas	Romania	Romania	Pharmacy	Food supplement
37	4	*Taraxacum officinale* Webb	Herbal teas	Romania	Romania	Pharmacy	Food supplement
38	4	*Achillea millefolium* L.	Herbal teas	Romania	Romania	Pharmacy	Food supplement
38	4	*Althaea officinalis* L.	Herbal teas	Romania	Romania	Pharmacy	Food supplement
38	4	*Convolvulus arvensis* L.	Herbal teas	Romania	Romania	Pharmacy	Food supplement
38	4	*Hypericum perforatum* L.	Herbal teas	Romania	Romania	Pharmacy	Food supplement
39	5	*Calendula officinalis* L.	Tablets	Romania	Romania	Pharmacy	Food supplement
39	5	*Foeniculum vulgare* Mill.	Tablets	Romania	Romania	Pharmacy	Food supplement
39	5	*Hypericum perforatum* L.	Tablets	Romania	Romania	Pharmacy	Food supplement
39	5	*Origanum vulgare* L.	Tablets	Romania	Romania	Pharmacy	Food supplement
39	5	Plantago spp.	Tablets	Romania	Romania	Pharmacy	Food supplement
40	2	*Hypericum perforatum* L.	Tablets	Romania	Romania	Pharmacy	Food supplement
40	2	*Lavandula latifolia* Medik. (syn. *Lavandula angustifolia* Moench)	Tablets	Romania	Romania	Pharmacy	Food supplement
41	1	*Hypericum perforatum* L.	Capsules	Romania	Romania	Pharmacy	Food supplement
42	7	*Aloe vera* (L.) Burm.f. (syn. *Aloe barbadensis* Mill.)	Capsules	Romania	Romania	Pharmacy	Food supplement
42	7	*Calendula officinalis* L.	Capsules	Romania	Romania	Pharmacy	Food supplement
42	7	*Citrus paradisi* Macfad.	Capsules	Romania	Romania	Pharmacy	Food supplement
42	7	*Hypericum perforatum* L.	Capsules	Romania	Romania	Pharmacy	Food supplement
42	7	*Lithothamnion calcareum* (Pallas)	Capsules	Romania	Romania	Pharmacy	Food supplement
42	7	*Melissa officinalis* L.	Capsules	Romania	Romania	Pharmacy	Food supplement
42	7	*Passiflora edulis* Sims (syn. *Passiflora incarnata* L.)	Capsules	Romania	Romania	Pharmacy	Food supplement
43	5	*Hypericum perforatum* L.	Capsules	Romania	Romania	Pharmacy	Food supplement
43	5	*Melissa officinalis* L.	Capsules	Romania	Romania	Pharmacy	Food supplement
43	5	*Passiflora edulis* Sims (syn. *Passiflora incarnata* L.)	Capsules	Romania	Romania	Pharmacy	Food supplement
43	5	*Trigonella foenum-graecum* L.	Capsules	Romania	Romania	Pharmacy	Food supplement
43	5	*Valeriana officinalis* L.	Capsules	Romania	Romania	Pharmacy	Food supplement
44	1	*Hypericum perforatum* L.	Herbal teas	Unknown	Spain	Health shop	Unknown
45	1	*Hypericum perforatum* L.	Herbal teas	Germany	Germany	Health shop	Herbal medicine
46	1	*Hypericum perforatum* L.	Herbal teas	Germany	Germany	Health shop	Herbal medicine
47	1	*Hypericum perforatum* L.	Capsules	Germany	Germany	Health shop	Herbal medicine
48	1	*Hypericum perforatum* L.	Tablets	Germany	Germany	Health shop	Herbal medicine
49	1	*Hypericum perforatum* L.	Tablets	France	France	Pharmacy	Herbal medicine
50	1	*Hypericum perforatum* L.	Herbal teas	Poland	Poland	E-commerce	Food supplement
51	2	*Hypericum perforatum* L.	Capsules	Poland	Poland	Health shop	Food supplement
51	2	Panax ginseng	Capsules	Poland	Poland	Health shop	Food supplement
52	11	*Avena sativa* L.	Herbal teas	Poland	Poland	Health shop	Herbal medicine
52	11	*Cirsium oleraceum* (L.) Scop.	Herbal teas	Poland	Poland	Health shop	Herbal medicine
52	11	*Citrus limon* (L.) Osbeck	Herbal teas	Poland	Poland	Health shop	Herbal medicine
52	11	Elsholtzia spp.	Herbal teas	Poland	Poland	Health shop	Herbal medicine
52	11	*Galium verum* L.	Herbal teas	Poland	Poland	Health shop	Herbal medicine
52	11	*Heracleum mantegazzianum* Sommier & Levier	Herbal teas	Poland	Poland	Health shop	Herbal medicine
52	11	*Humulus lupulus* L.	Herbal teas	Poland	Poland	Health shop	Herbal medicine
52	11	*Hypericum perforatum* L.	Herbal teas	Poland	Poland	Health shop	Herbal medicine
52	11	*Stachys officinalis* (L.) Trevis.	Herbal teas	Poland	Poland	Health shop	Herbal medicine
52	11	*Verbena officinalis* L.	Herbal teas	Poland	Poland	Health shop	Herbal medicine
52	11	*Veronica officinalis* L.	Herbal teas	Poland	Poland	Health shop	Herbal medicine
53	1	*Hypericum perforatum* L.	Tablets	Poland	Poland	Health shop	Herbal medicine
54	2	*Hypericum perforatum* L.	Capsules	Italy	Italy	Health shop	Food supplement
54	2	Tilia spp.	Capsules	Italy	Italy	Health shop	Food supplement
55	1	*Hypericum perforatum* L.	Herbal teas	USA	Czech Republic	Health shop	Food supplement
56	1	*Hypericum perforatum* L.	Herbal teas	Austria	Austria	Pharmacy	Unknown
57	9	*Achillea millefolium* L.	Herbal teas	Austria	Austria	Pharmacy	Unknown
57	9	*Elymus repens* (L.) Gould	Herbal teas	Austria	Austria	Pharmacy	Unknown
57	9	*Glycyrrhiza glabra* L.	Herbal teas	Austria	Austria	Pharmacy	Unknown
57	9	*Hypericum perforatum* L.	Herbal teas	Austria	Austria	Pharmacy	Unknown
57	9	*Juniperus communis* L.	Herbal teas	Austria	Austria	Pharmacy	Unknown
57	9	*Mentha × piperita* L.	Herbal teas	Austria	Austria	Pharmacy	Unknown

57	9	*Pimpinella anisum* L.	Herbal teas	Austria	Austria	Pharmacy	Unknown
57	9	*Taraxacum officinale* Webb	Herbal teas	Austria	Austria	Pharmacy	Unknown
57	9	*Viola tricolor* L.	Herbal teas	Austria	Austria	Pharmacy	Unknown
58	1	*Hypericum perforatum* L.	Herbal teas	Sweeden	Sweeden	Health shop	Herbal medicine
59	1	*Hypericum perforatum* L.	Extracts	Germany	Germany	Health shop	Unknown
60	1	*Hypericum perforatum* L.	Tablets	USA	Nederland	Health shop	Food supplement
61	1	*Hypericum perforatum* L.	Tablets	Unknown	UK	Health shop	Herbal medicine
62	5	*Citrus limon* (L.) Osbeck	Herbal teas	Slovakia	United Kingdom	Health shop	Food supplement
62	5	*Citrus sinensis* (L.) Osbeck	Herbal teas	Slovakia	Slovakia	Health shop	Food supplement
62	5	*Hypericum perforatum* L.	Herbal teas	Slovakia	Slovakia	Health shop	Food supplement
62	5	Lavandula spp.	Herbal teas	Slovakia	Slovakia	Health shop	Food supplement
62	5	*Passiflora edulis* Sims (syn. *Passiflora incarnata* L.)	Herbal teas	Slovakia	Slovakia	Health shop	Food supplement
63	4	*Humulus lupulus* L.	Herbal teas	Czech Republic	Slovakia	Health shop	Food supplement
63	4	*Hypericum perforatum* L.	Herbal teas	Czech Republic	Slovakia	Health shop	Food supplement
63	4	*Matricaria chamomilla* L.	Herbal teas	Czech Republic	Slovakia	Health shop	Food supplement
63	4	Mentha spp.	Herbal teas	Czech Republic	Slovakia	Health shop	Food supplement
63	4	*Valeriana officinalis* L.	Herbal teas	Czech Republic	Slovakia	Health shop	Food supplement
64	10	*Achillea millefolium* L.	Herbal teas	Slovakia	Slovakia	Health shop	Food supplement
64	10	*Acorus calamus* var. americanus Raf.	Herbal teas	Slovakia	Slovakia	Health shop	Food supplement
64	10	*Calendula officinalis* L.	Herbal teas	Slovakia	Slovakia	Health shop	Food supplement
64	10	*Centaurium erythraea* Rafn	Herbal teas	Slovakia	Slovakia	Health shop	Food supplement
64	10	*Cichorium intybus* L.	Herbal teas	Slovakia	Slovakia	Health shop	Food supplement
64	10	*Galium verum* L.	Herbal teas	Slovakia	Slovakia	Health shop	Food supplement
64	10	*Helichrysum arenarium* (L.) DC.	Herbal teas	Slovakia	Slovakia	Health shop	Food supplement
64	10	*Hypericum perforatum* L.	Herbal teas	Slovakia	Slovakia	Health shop	Food supplement
64	10	*Taraxacum officinale* Webb	Herbal teas	Slovakia	Slovakia	Health shop	Food supplement
64	10	*Urtica dioica* L.	Herbal teas	Slovakia	Slovakia	Health shop	Food supplement
65	1	*Hypericum perforatum* L.	Tablets	United Kingdom	United Kingdom	Health shop	Herbal medicine
66	1	*Hypericum perforatum* L.	Capsules	Spain	Spain	Pharmacy	Herbal medicine
67	1	*Hypericum perforatum* L.	Tablets	USA	Turkey	Pharmacy	Food supplement
68	2	*Hypericum perforatum* L.	Extracts	Turkey	Turkey	Pharmacy	Unknown
68	2	*Olea europaea* L.	Extracts	Turkey	Turkey	Pharmacy	Unknown
69	1	*Hypericum perforatum* L.	Extracts	Turkey	Turkey	Pharmacy	Unknown
70	13	*Achillea millefolium* L.	Herbal teas	Turkey	Turkey	Pharmacy	Unknown
70	13	*Calluna vulgaris* (L.) Hull	Herbal teas	Turkey	Turkey	Pharmacy	Unknown
70	13	*Camellia sinensis* (L.) Kuntze	Herbal teas	Turkey	Turkey	Pharmacy	Unknown
70	13	*Foeniculum vulgare* Mill.	Herbal teas	Turkey	Turkey	Pharmacy	Unknown
70	13	*Frangula dodonei* Ard.(*Rhamnus frangula* L.)	Herbal teas	Turkey	Turkey	Pharmacy	Unknown
70	13	Hibiscus spp.	Herbal teas	Turkey	Turkey	Pharmacy	Unknown
70	13	*Hypericum perforatum* L.	Herbal teas	Turkey	Turkey	Pharmacy	Unknown
70	13	*Paullinia cupana* Kunth	Herbal teas	Turkey	Turkey	Pharmacy	Unknown
70	13	*Pimpinella anisum* L.	Herbal teas	Turkey	Turkey	Pharmacy	Unknown
70	13	*Prunus avium* (L.) L.	Herbal teas	Turkey	Turkey	Pharmacy	Unknown
70	13	*Rosmarinus officinalis* L.	Herbal teas	Turkey	Turkey	Pharmacy	Unknown
70	13	*Satureja hortensis* L.	Herbal teas	Turkey	Turkey	Pharmacy	Unknown
70	13	*Stevia rebaudiana* (Bertoni) Bertoni	Herbal teas	Turkey	Turkey	Pharmacy	Unknown
71	8	*Achillea millefolium* L.	Herbal teas	Romania	Romania	Pharmacy	Food supplement
71	8	*Calendula officinalis* L.	Herbal teas	Romania	Romania	Pharmacy	Food supplement
71	8	*Gentiana asclepiadea* L.	Herbal teas	Romania	Romania	Pharmacy	Food supplement
71	8	*Hypericum perforatum* L.	Herbal teas	Romania	Romania	Pharmacy	Food supplement
71	8	*Melissa officinalis* L.	Herbal teas	Romania	Romania	Pharmacy	Food supplement
71	8	*Silybum marianum (L.) Gaertn.* (syn. *Carduus marianus L.)*	Herbal teas	Romania	Romania	Pharmacy	Food supplement
71	8	*Stevia rebaudiana* (Bertoni) Bertoni	Herbal teas	Romania	Romania	Pharmacy	Food supplement
71	8	*Veronica officinalis* L.	Herbal teas	Romania	Romania	Pharmacy	Food supplement
72	6	*Cichorium intybus* L.	Capsules	Romania	Romania	Pharmacy	Food supplement
72	6	*Cynara scolymus* L.	Capsules	Romania	Romania	Pharmacy	Food supplement
72	6	*Gentiana lutea* L.	Capsules	Romania	Romania	Pharmacy	Food supplement
72	6	*Hypericum perforatum* L.	Capsules	Romania	Romania	Pharmacy	Food supplement
72	6	*Silybum marianum* (L.) Gaertn.	Capsules	Romania	Romania	Pharmacy	Food supplement
72	6	*Taraxacum officinale* Webb	Capsules	Romania	Romania	Pharmacy	Food supplement
73	11	*Achillea millefolium* L.	Extracts	Romania	Romania	Pharmacy	Food supplement
73	11	*Calendula officinalis* L.	Extracts	Romania	Romania	Pharmacy	Food supplement
73	11	*Carthamus tinctorius* L.	Extracts	Romania	Romania	Pharmacy	Food supplement
73	11	*Chelidonium majus* L.	Extracts	Romania	Romania	Pharmacy	Food supplement
73	11	*Cynara scolymus* L.	Extracts	Romania	Romania	Pharmacy	Food supplement
73	11	*Gentiana lutea* L.	Extracts	Romania	Romania	Pharmacy	Food supplement
73	11	*Hypericum perforatum* L.	Extracts	Romania	Romania	Pharmacy	Food supplement
73	11	*Lavandula angustifolia subsp. angustifolia* (syn. *Lavandula officinalis* Chaix)	Extracts	Romania	Romania	Pharmacy	Food supplement
73	11	*Melissa officinalis* L.	Extracts	Romania	Romania	Pharmacy	Food supplement
73	11	*Silybum marianum* (L.) Gaertn.	Extracts	Romania	Romania	Pharmacy	Food supplement
73	11	*Viscum album* L.	Extracts	Romania	Romania	Pharmacy	Food supplement
74	10	*Agrimonia eupatoria* L.	Herbal teas	Romania	Romania	Health shop	Food supplement
74	10	*Calendula officinalis* L.	Herbal teas	Romania	Romania	Health shop	Food supplement
74	10	*Centaurium erythraea* Rafn	Herbal teas	Romania	Romania	Health shop	Food supplement
74	10	*Chelidonium majus* L.	Herbal teas	Romania	Romania	Health shop	Food supplement
74	10	*Cichorium intybus* L.	Herbal teas	Romania	Romania	Health shop	Food supplement
74	10	*Cynara scolymus* L.	Herbal teas	Romania	Romania	Health shop	Food supplement
74	10	*Equisetum arvense* L.	Herbal teas	Romania	Romania	Health shop	Food supplement
74	10	*Gentiana lutea* L.	Herbal teas	Romania	Romania	Health shop	Food supplement
74	10	*Hypericum perforatum* L.	Herbal teas	Romania	Romania	Health shop	Food supplement
74	10	*Lycopodium clavatum* L.	Herbal teas	Romania	Romania	Health shop	Food supplement
75	18	*Achillea millefolium* L.	Herbal teas	Romania	Romania	Health shop	Food supplement
75	18	*Agrimonia eupatoria* L.	Herbal teas	Romania	Romania	Health shop	Food supplement
75	18	*Artemisia absinthium* L.	Herbal teas	Romania	Romania	Health shop	Food supplement
75	18	*Calendula officinalis* L.	Herbal teas	Romania	Romania	Health shop	Food supplement
75	18	*Calendula officinalis* L.	Herbal teas	Romania	Romania	Health shop	Food supplement
75	18	*Centaurium erythraea* Rafn	Herbal teas	Romania	Romania	Health shop	Food supplement
75	18	*Cotinus coggygria* Scop.	Herbal teas	Romania	Romania	Health shop	Food supplement
75	18	*Echinacea* spp.	Herbal teas	Romania	Romania	Health shop	Food supplement
75	18	*Equisetum arvense* L.	Herbal teas	Romania	Romania	Health shop	Food supplement
75	18	*Gentiana lutea* L.	Herbal teas	Romania	Romania	Health shop	Food supplement
75	18	*Geranium robertianum* L.	Herbal teas	Romania	Romania	Health shop	Food supplement
75	18	*Hypericum perforatum* L.	Herbal teas	Romania	Romania	Health shop	Food supplement
75	18	*Linaria vulgaris* Mill.	Herbal teas	Romania	Romania	Health shop	Food supplement
75	18	*Lythrum salicaria* L.	Herbal teas	Romania	Romania	Health shop	Food supplement
75	18	*Robinia pseudoacacia* L.	Herbal teas	Romania	Romania	Health shop	Food supplement
75	18	*Salvia officinalis* L.	Herbal teas	Romania	Romania	Health shop	Food supplement
75	18	*Symphytum officinale* L.	Herbal teas	Romania	Romania	Health shop	Food supplement

75	18	*Zea mays* L.	Herbal teas	Romania	Romania	Health shop	Food supplement
76	18	*Achillea millefolium* L.	Herbal teas	Romania	Romania	Health shop	Food supplement
76	18	*Agrimonia eupatoria* L.	Herbal teas	Romania	Romania	Health shop	Food supplement
76	18	*Antirrhinum majus* L.	Herbal teas	Romania	Romania	Health shop	Food supplement
76	18	*Aruncus dioicus* (Walter) Fernald	Herbal teas	Romania	Romania	Health shop	Food supplement
76	18	*Calendula officinalis* L.	Herbal teas	Romania	Romania	Health shop	Food supplement
76	18	*Centaurium erythraea* Rafn	Herbal teas	Romania	Romania	Health shop	Food supplement
76	18	*Chelidonium majus* L.	Herbal teas	Romania	Romania	Health shop	Food supplement
76	18	*Cichorium intybus* L.	Herbal teas	Romania	Romania	Health shop	Food supplement
76	18	*Cynara scolymus* L.	Herbal teas	Romania	Romania	Health shop	Food supplement
76	18	Echinacea spp.	Herbal teas	Romania	Romania	Health shop	Food supplement
76	18	*Equisetum arvense* L.	Herbal teas	Romania	Romania	Health shop	Food supplement
76	18	*Gentiana lutea* L.	Herbal teas	Romania	Romania	Health shop	Food supplement
76	18	*Hypericum perforatum* L.	Herbal teas	Romania	Romania	Health shop	Food supplement
76	18	*Linaria vulgaris* Mill.	Herbal teas	Romania	Romania	Health shop	Food supplement
76	18	*Lycopodium clavatum* L.	Herbal teas	Romania	Romania	Health shop	Food supplement
76	18	*Potentilla anserina* L. (syn. *Argentina anserina* (L.) Rydb.)	Herbal teas	Romania	Romania	Health shop	Food supplement
76	18	*Salvia officinalis* L.	Herbal teas	Romania	Romania	Health shop	Food supplement
76	18	*Verbascum phlomoides* L.	Herbal teas	Romania	Romania	Health shop	Food supplement
77	23	*Achillea millefolium* L.	Herbal teas	Romania	Romania	Health shop	Food supplement
77	23	*Agrimonia eupatoria* L.	Herbal teas	Romania	Romania	Health shop	Food supplement
77	23	*Antirrhinum majus* L.	Herbal teas	Romania	Romania	Health shop	Food supplement
77	23	*Asplenium scolopendrium* var. *americanum* (Fernald) Kartesz & Gandhi	Herbal teas	Romania	Romania	Health shop	Food supplement
77	23	*Calendula officinalis* L.	Herbal teas	Romania	Romania	Health shop	Food supplement
77	23	*Centaurium erythraea* Rafn (syn. *Centaurium umbellatum* Gilib.)	Herbal teas	Romania	Romania	Health shop	Food supplement
77	23	*Chelidonium majus* L.	Herbal teas	Romania	Romania	Health shop	Food supplement
77	23	*Cichorium intybus* L.	Herbal teas	Romania	Romania	Health shop	Food supplement
77	23	*Cynara scolymus* L.	Herbal teas	Romania	Romania	Health shop	Food supplement
77	23	*Echinacea purpurea* (L.) Moench	Herbal teas	Romania	Romania	Health shop	Food supplement
77	23	*Equisetum arvense* L.	Herbal teas	Romania	Romania	Health shop	Food supplement
77	23	*Filipendula ulmaria* (L.) Maxim.	Herbal teas	Romania	Romania	Health shop	Food supplement
77	23	*Frangula dodonei* Ard.(*Rhamnus frangula* L.)	Herbal teas	Romania	Romania	Health shop	Food supplement
77	23	*Gentiana lutea* L.	Herbal teas	Romania	Romania	Health shop	Food supplement
77	23	*Gentiana punctata* L.	Herbal teas	Romania	Romania	Health shop	Food supplement
77	23	*Hypericum perforatum* L.	Herbal teas	Romania	Romania	Health shop	Food supplement
77	23	*Linaria vulgaris* Mill.	Herbal teas	Romania	Romania	Health shop	Food supplement
77	23	*Lycopodium clavatum* L.	Herbal teas	Romania	Romania	Health shop	Food supplement
77	23	*Lysimachia vulgaris* L.	Herbal teas	Romania	Romania	Health shop	Food supplement
77	23	*Potentilla anserina* L.	Herbal teas	Romania	Romania	Health shop	Food supplement
77	23	*Salvia officinalis* L.	Herbal teas	Romania	Romania	Health shop	Food supplement
77	23	*Verbascum phlomoides* L.	Herbal teas	Romania	Romania	Health shop	Food supplement
78	7	*Achillea millefolium* L.	Extracts	Romania	Romania	Pharmacy	Food supplement
78	7	*Calendula officinalis* L.	Extracts	Romania	Romania	Pharmacy	Food supplement
78	7	*Gentiana lutea* L.	Extracts	Romania	Romania	Pharmacy	Food supplement
78	7	*Hypericum perforatum* L.	Extracts	Romania	Romania	Pharmacy	Food supplement
78	7	*Melissa officinalis* L.	Extracts	Romania	Romania	Pharmacy	Food supplement
78	7	*Silybum marianum* (L.) Gaertn.	Extracts	Romania	Romania	Pharmacy	Food supplement
78	7	*Veronica officinalis* L.	Extracts	Romania	Romania	Pharmacy	Food supplement
